# Establishment and maintenance of embryogenic cell fate during microspore embryogenesis

**DOI:** 10.1111/tpj.17243

**Published:** 2025-02-21

**Authors:** Charlotte Siemons, Sven Jonkers, Redmar Cornelis Vlieg, Patricia Corral‐Martínez, John van Noort, Kim Boutilier

**Affiliations:** ^1^ Bioscience Wageningen University & Research P.O. Box 16 6700 AA Wageningen The Netherlands; ^2^ Laboratory of Molecular Biology Wageningen University & Research P.O. Box 633 6700 AP Wageningen The Netherlands; ^3^ Enza Zaden Haling 1 1602 DB Enkhuizen The Netherlands; ^4^ Biological and Soft Matter Physics, Huygens‐Kamerlingh Onnes Laboratory Leiden University Niels Bohrweg 2 2333 CA Leiden The Netherlands; ^5^ Present address: University of Utrecht Universiteitsweg 99 3584 CG Utrecht The Netherlands

**Keywords:** auxin, *Brassica napus*, *DR5v2*, cell division plane, cell fate, *LEAFY COTYLEDON1*, microspore embryogenesis, totipotency

## Abstract

Microspore embryogenesis is a type of *in vitro* totipotency in which the immature male gametophyte (pollen) develops into a haploid embryo after an abiotic stress treatment. In *Brassica napus*, heat‐stress treatment of male gametophytes induces the development of different types of multicellular embryogenic structures, each with different cellular characteristics and the capacity to form a differentiated embryo. The origin and early development of these different embryogenic structures have not been determined. We used two‐photon excitation fluorescence microscopy and time‐lapse imaging of cells expressing either a *LEAFY COTYLEDON1* (*LEC1*) embryo identity reporter or a *DR5v2* auxin response reporter to follow the development of embryogenic structures starting at the single‐ to few‐cell stage. We show for the first time that the developmental fate of embryogenic structures is defined by the symmetry of the first embryogenic division and that the division plane also predicts the timing of subsequent pollen wall (exine) rupture: suspensorless embryos develop after a symmetric division and undergo late exine rupture, while suspensor‐bearing embryos and embryogenic callus develop after an asymmetric division and undergo early exine rupture. Live imaging also captured previously unknown dynamic *LEC1* and *DR5v2* expression patterns that are associated with changes in exine integrity. This study highlights the developmental plasticity of cultured pollen and uncovers new roles for the first embryogenic cell division plane and the exine in defining and maintaining cell fate during microspore embryogenesis.

## Significance Statement

The origin and early development of *in vitro* cultured embryos are enigmatic, in part, due to the use of static imaging techniques that only provide a single snapshot of development in time. We used time‐lapse imaging of embryo identity and auxin response reporter lines to track cultured *Brassica napus* pollen as they develop into multicellular embryogenic structures, thereby resolving long‐standing questions about the origin and fate of haploid embryogenic cells.

## INTRODUCTION

In sexually reproducing plants, embryogenesis begins with the fusion of the haploid egg and sperm cells to form the diploid zygote and culminates in the formation of a differentiated embryo with the meristems and tissue progenitor cells required for post‐germination growth of the plant. Many plant cells can develop into embryos in the absence of fertilization, either naturally *in planta* or after induction *in vitro* (León‐Martínez & Vielle‐Calzada, 2019; Shen et al.,^2023^; Tian et al., 2020). Microspore embryogenesis is a type of totipotency in which *in vitro* cultured immature male gametophytes (microspores and pollen) are induced by an abiotic stress treatment to change developmental fate and develop into haploid embryos. Microspore embryogenesis is a widely applied plant breeding technique that is used to develop segregating populations of homozygous “doubled‐haploid” plants in a single generation (Hale et al., 2021), but it is also used as a model system to understand the mechanism driving plant cell totipotency (Testillano, 2019).

Microspore embryogenesis from isolated *Brassica napus (B. napus)* male gametophytes was first described in the early 1980s (Lichter, 1982). Heat‐stress (HS) treatment of isolated microspores is used to induce *B. napus* microspore embryogenesis, but embryogenic cell formation and differentiated embryo yield and quality can be greatly enhanced in *B. napus* and/or other species by chemical inhibition of histone/lysine deacetylase activity, for example, by trichostatin A (TSA) (Castillo et al., 2020; Corral‐Martínez et al., 2020; Jiang et al., 2017; Li et al., 2014).

Zygotic embryogenesis in *B. napus* and the related model plant *Arabidopsis thaliana* (arabidopsis) follows a series of highly predictable cell divisions (ten Hove et al., 2015; Tykarska, 1976, 1979). The zygote divides asymmetrically to form two daughter cells with different developmental fates: a smaller apical cell and a larger vacuolated basal cell, which together form the basis for the main apical‐basal body axis of the embryo. The apical cell generates most of the cells of the embryo proper, while the basal cell contributes to the hypophysis of the embryo proper and to the suspensor. *B. napus* microspore embryo development is more flexible than zygotic embryogenesis, and at least four different types of embryogenic development have been described (Corral‐Martínez et al., 2020; Li et al., 2014). These different embryogenic pathways can be distinguished morphologically around 4–5 days after the start of microspore culture (Figure S1). The first two embryo development pathways lead to the development of differentiated embryos with or without a suspensor. Unlike zygotic embryos, suspensorless embryos lack an obvious suspensor but still have a well‐developed root pole (Figure S1A,E; Yeung et al., 1996). Suspensorless embryos develop within the original pollen wall (exine) until the multicellular globular stage, at which point the exine stretches and then ruptures. Apical‐basal patterning and tissue differentiation are initiated after exine rupture. Suspensor embryos resemble zygotic embryos in that they compromise an apical embryo proper and a basal suspensor filament or suspensor‐like protrusion (Figure S1B,F). Suspensor embryos are recognizable as loosely connected, few‐celled structures with a clear apical‐basal axis, and by their larger cells and thicker cell walls than suspensorless embryos. Exine rupture takes place earlier than in the suspensorless embryo pathway, starting around the two‐cell stage, and usually occurs parallel to the first division plane. The other two embryo development pathways lead to the formation of compact or loose embryogenic callus. Embryogenic calli are multicellular structures (up to 10 cells) that undergo exine rupture early, around the two‐cell stage, and do not have a clear apical‐basal axis (Figure S1C,D,G,H). Embryogenic calli show more extensive exine rupture and have the least cell adhesion, the largest cells, and the thickest walls of all embryogenic structures. Loose callus shows more extreme loss of cell adhesion than compact callus, often with complete cell separation. The vast majority of calli are unable to form differentiated embryos, although loose calli occasionally develop into suspensor embryos (Corral‐Martínez et al., 2013). In *B. napus* zygotic embryos, *LEC1* is expressed in the suspensor and the embryo proper (Li et al., 2014). The *LEC1:LEC1‐GFP* embryo identity reporter is expressed from the single‐cell stage onwards in microspore embryo cultures, and later its expression marks all the above embryo development pathways (Figure S1A–D; Li et al., 2014). The *LEC1* reporter is not expressed in the pollen that also develop in microspore culture (Li et al., 2014).

The developmental flexibility of microspore embryos is also reflected in their auxin responses. The phytohormone auxin influences almost every aspect of plant development and growth and likewise plays an important role in zygotic embryo patterning. *DR5*‐based auxin response reporters are used as indirect read‐outs of auxin levels and/or signaling (Liao et al., 2015; Ulmasov et al., 1997). In Arabidopsis zygotic embryos, *DR5* reporter activity is initially localized to the embryo proper and then shifts to the hypophysis and upper suspensor cell at the globular stage (Friml et al., 2003; Liao et al., 2015). In *B. napus* microspore embryo cultures, *DR5rev* activity transiently marks a subset of single‐ and two‐cell structures of unknown fate (Soriano et al., 2014). Later, the *DR5rev* reporter is strongly expressed in future suspensorless globular‐stage embryos that show stretching/rupture of the exine and then becomes restricted to a few cells that mark the site of the presumptive root meristem fate (Soriano et al., 2014). *DR5rev* expression in suspensor embryos is similar to that in zygotic embryos, with expression being restricted to the embryo proper. *DR5rev* activity is not observed in embryogenic callus in 4‐ to 5‐day‐old cultures, nor is it observed in the pollen cells that also develop in microspore culture (Soriano et al., 2014).

Previously, we used time‐lapse imaging of a *B. napus LEC1:LEC1‐GFP* embryo identity reporter line to track the fate of the different types of multicellular embryogenic structures starting on day 5 of microspore culture, which is the time point at which the different types of embryogenic structures can be morphologically distinguished (Corral‐Martínez et al., 2020). Here, we used two‐photon excitation fluorescence microscopy (TPEFM) to follow cell fate (*LEC1:LEC1‐GFP*) and auxin response (*DR5v2:ntdTomato*) in single‐ to few‐celled embryogenic structures 24 h after the start of culture. This approach allowed us to define the role of the first embryogenic division plane in the developmental fate of embryogenic cells and its relation to the timing of exine rupture, as well as the role of the exine in maintaining embryo identity and auxin response.

## RESULTS

### Time‐lapse imaging system

Previously, we attempted to follow the developmental fate of immobilized microspores using CLSM directly after the 24‐h HS + TSA treatment (Corral‐Martínez et al., 2020), but both the immobilization in agarose and the imaging system had a negative effect on the progression of microspore embryogenesis, with the result that this system could only be used to track the development of microspore embryo cultures from day 5 of culture (Corral‐Martínez et al., 2020). Here, we used the same agarose‐based system to immobilize the donor microspores/pollen (Figure S2A) but increased the cell density from 1.0 × 10^6^ to 5.0 × 10^6^ microspores mL^−1^. We determined the percentage of embryogenic divisions and the different types of embryogenic structures that developed in immobilized cultures and control cultures without immobilization (Figure S2B,C). Both cultures were pretreated in 50 mL tubes for 24 h with HS + 0.05 μM TSA to induce ME. We did not observe any statistically significant difference in the proportion of embryogenic divisions or the types of embryogenic structures in the control and immobilized cultures. Next, we evaluated the effect of our custom TPEFM imaging system on the progression of ME. Unlike our previous CLSM time‐lapse study, TPEFM imaging did not affect the percentage of embryogenic structures that developed in immobilized cultures (Figure S2D), nor did we observe the abnormally large calli that were seen previously using CLSM (Corral‐Martínez et al., 2020). Thus, embedding microspores/pollen at a high density and imaging with a custom TPEFM system allowed us to image microspore embryo structures from day 2 of culture without compromising their viability or altering their development.

Using this system, we selected and successfully tracked the development of 86 *LEC1*‐expressing cells/multicellular structures in a *LEC1:LEC1‐GFP* line and 39 *DR5v2*‐expressing cells/multicellular structures in a *DR5v2:ntdTomato‐*line that were present at the start of the tracking period. Embryogenic structures with one to four cells were observed at the start of the tracking period but could not be classified as suspensorless embryos, suspensor embryos, or embryogenic calli. Therefore, the developmental fate of each tracked cell/structure was established at the end of the tracking period based on morphology. The cellular origin and orientation of the first embryogenic division plane were either observed during tracking in *LEC1:LEC1‐GFP* or *DR5v2:ntdTomato‐*expressing microspores or inferred from *LEC1:LEC1‐GFP* or *DR5v2:ntdTomato‐*expressing two‐celled structures present at the start of tracking.

### Exine‐eclosed structures with limited cell divisions

Several *LEC1:LEC1‐GFP* or *DR5v2:ntdTomato‐*expressing structures (*n* = 19) stopped dividing after a few cell divisions and did not undergo exine rupture (Table 1; Figure S3). These structures continued to express the *LEC1* reporter during the tracking period, but gradually lost expression of the *DR5v2:ntdTomato* reporter (Table 1). These data suggest that embryo identity can be maintained in the absence of sustained cell division and in the absence of a *DR5* auxin response.

**Table 1:** Developmental fates of tracked *LEC1* (*LEC1:LEC1‐GFP*) and *DR5v2* (*DR5v2:ntdTomato*)‐expressing embryogenic structures. Expressing structures were identified at the start of the tracking period and then tracked for 4–6 days. The number of tracked structures (*n*) and percentages of the totals in each category are shown

	Final fate (*n*)	Limited divisions (20/16%)		Suspensorless embryo (48/38.4%)		Suspensor embryo (8/6.4%)		Embryogenic callus (49/39.2%)	
	Early events	No exine rupture		Exine rupture at day 4		Exine rupture at day 3		Exine rupture at days 1–3	
*LEC1:LEC‐GFP* expression	*n* = 86	Expression	Loss of expression	Expression	Loss of expression	Expression and sustained division	Loss of expression and division arrest	Expression and sustained division	Loss of expression and division arrest
Initial developmental pathway	Exine‐enclosed (8)	8 (9%)							
	Suspensorless (43)			20 (23%)	21 (24%)			2 (2.3%)	
	Suspensor (8)	1 (1.7%)				6 (8.0%)		1 (1.7%)	
	Callus (27)								27 (31.4%)
*DR5v2:ntdTomato* expression	*n* = 39	Expression	Loss of expression	Expression	Loss of expression	Expression and sustained division	Loss of expression and division arrest	Expression and sustained division	Loss of expression and division arrest
Initial developmental pathway	Exine‐enclosed (11)		11 (28.2%)						
	Suspensorless (7)			3 (7.7%)	4 (10.3%)				
	Suspensor (2)					2 (5.1%)			
	Callus (19)								19 (48.7%)

### Suspensorless embryo pathway

**Figure 1:**
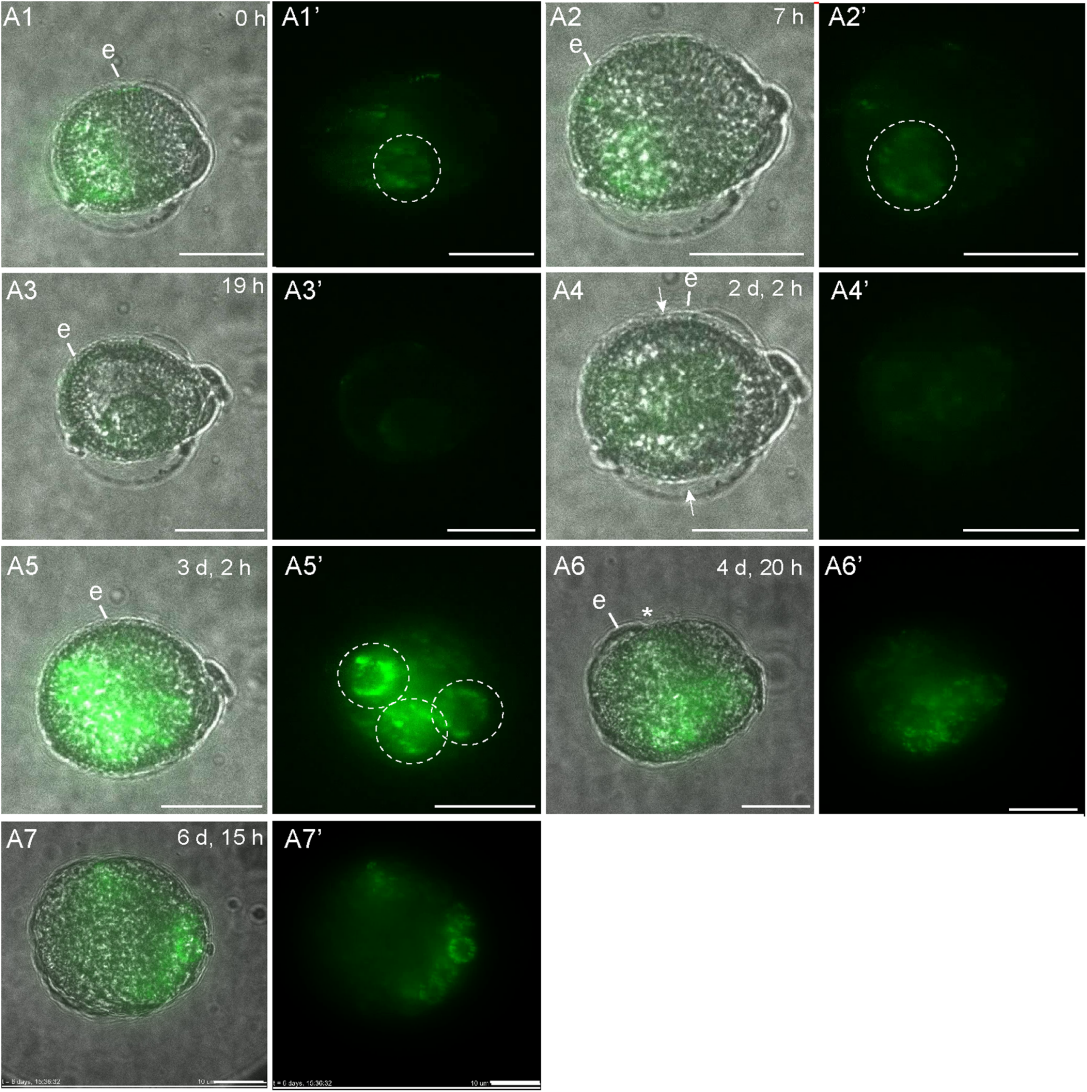
*LEC1:LEC1‐GFP* expression during suspensorless embryo development. The single‐cell microspore moved from a lateral position (A1–A3) to a central position (A4), where it divided symmetrically to form two equal‐sized cells (A4). A transient reduction in *LEC1:LEC1‐GFP* expression was observed at the one‐ and two‐cell stages (A3, A4). The exine started to burst around 4 days after the start of tracking (A6). A transmission image is shown next to the fluorescence image for each timepoint. The green signal corresponds to GFP fluorescence. All images were autoscaled to reduce the fluorescence intensity. White arrows indicate the first embryogenic cell division plane; white dashed circles indicate nuclear *LEC1* nuclear expression; e, exine; *, site of exine rupture. Scale bar = 10 μm. The non‐autoscaled videos used for this figure can be found in Supporting Information.

**Figure 2:**
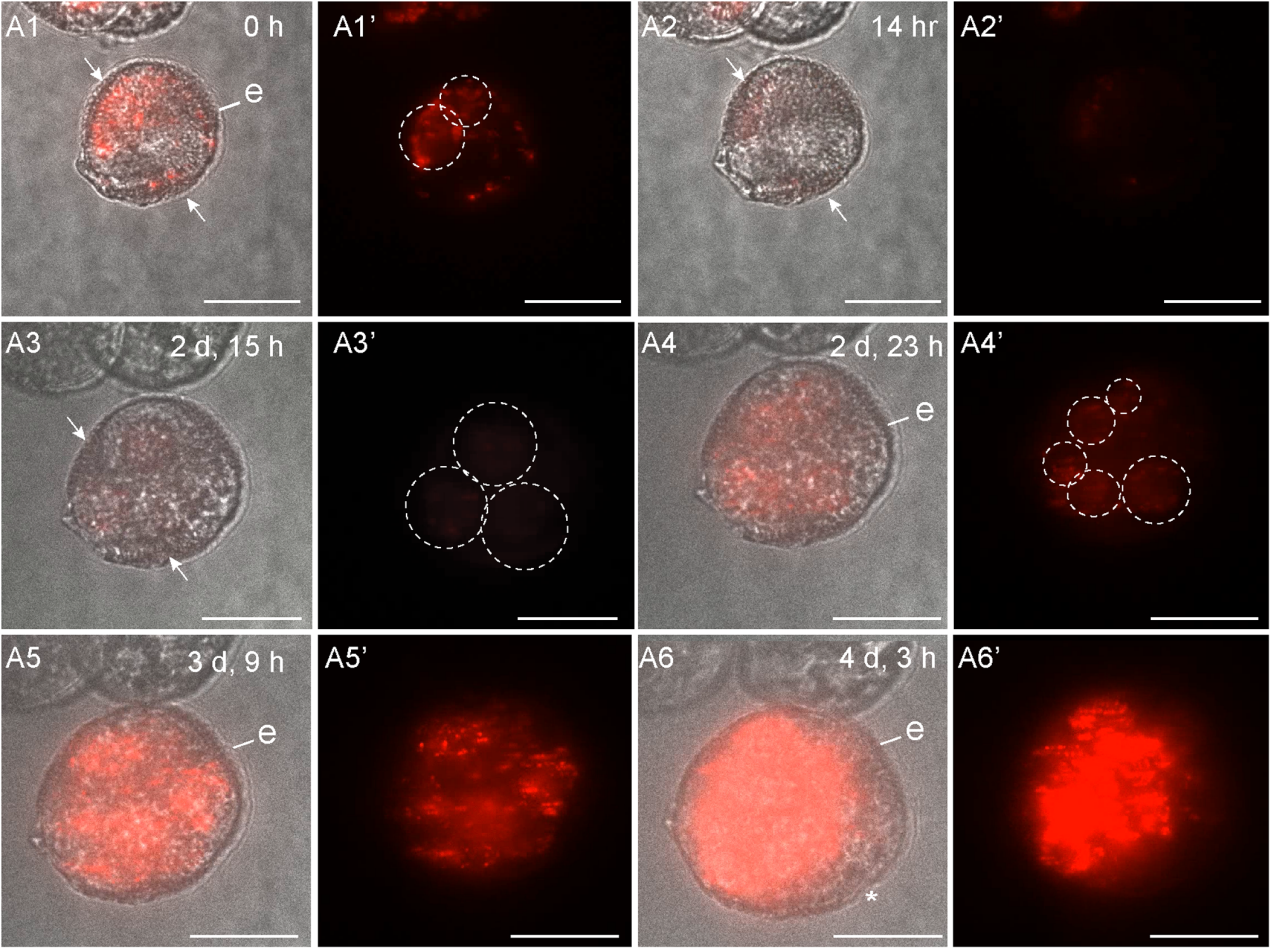
*DR5v2:ntdTomato* expression during suspensorless embryo development. The embryo developed after an initial symmetric division (A1), as inferred from the position of the nuclei in the two‐celled structure. *DR5v2:ntdTomato* expression transiently decreased during the two‐celled stage (A2, A3), followed by an increase in expression at the multicellular stage (A4, A5). Exine rupture started on day 4 of tracking (A6). For each time point, a transmission image is shown next to the fluorescence image. The red signal corresponds to tdTomato fluorescence. White arrows indicate the first embryogenic cell division plane; white dashed circles indicate *DR5v2* nuclear expression; e, exine; asterisk (*), site of exine rupture. Scale bar = 10 μm. The videos used for this figure can be found in Supporting Information.

Of the 125 structures showing either *LEC1:LEC1:GFP* or *DR5v2:ntdTomato* reporter expression at the start of tracking, 48 developed into suspensorless embryos, that is, multicellular globular embryos that underwent relatively late exine rupture (Table 1). The vast majority of suspensorless embryos developed after symmetric division of *LEC1* or *DR5v2*‐expressing cells (Table 2; Figures 1A4 and 2A1; Figures S4A3 and S5A1). When microspore division was observed, in most instances (*n* = 4/5), the nucleus first migrated from a peripheral to central position (Figure 1A1–A4). This first symmetric division generated two large nuclei (Figure 2A1; Figures S4A3 and S5A1), rather than the larger vegetative and smaller generative cell that are formed after division of the microspore into a bicellular pollen at pollen mitosis I (Figure S1K). Most embryogenic structures that developed into suspensorless embryos already had two cells at the start of tracking (Table 2). We did not include a generative cell reporter in this study; therefore, for these inferred divisions, we could not determine whether the microspore (Figure S1I,K) or vegetative cell of a bicellular pollen divided symmetrically (Figure S1K). Symmetric division of both the microspore and the vegetative cell has been observed in *B. napus* using static imaging (Fan et al., 1988; Zaki & Dickinson, 1991). Suspensorless embryos underwent exine rupture around day 4–6 of tracking (Figures 1A6 and 2A6; Figures S4A8 and S5A5).

**Table 2:** Type of first embryogenic cell division observed in LEC1 and DR5v2‐expressing cells with different developmental fates. The cell division plane was either observed in LEC1 or DR5v2‐expressing microspores that divided during the tracking period or was inferred from LEC1 or DR5v2‐expressing two‐celled structures that were present at the start of the tracking period, that is, embryogenic structures that had already divided in the 24 h prior to tracking. The cell division plane (symmetric/asymmetric) is indicated. The developmental fate is indicated for each structure. LEC1 or DR5v2‐expressing structures that did not divide or only divided a few times but did not show exine rupture (see Table 1) are not included, as the final fate of these structures is not known. The total number of observed structures is shown. The numbers in brackets indicate the number of tracked LEC1 or DR5v2‐expressing structures, respectively

	Developmental fate					
	Suspensorless embryo (*LEC1* + *DR5v2*)		Suspensor embryo (*LEC1* + *DR5v2*)		Embryogenic callus (*LEC1* + *DR5v2*)	
Division/observed in	Microspore	2‐cell structure	Microspore	2‐cell structure	Microspore	2‐cell structure
Symmetric	5 (4 + 1)	15 (12 + 3)	0	0	1 (0 + 1)	2 (1 + 1)
Asymmetric	1 (1 + 0)	0	1 (0 + 1)	6 (4 + 2)	5 (1 + 4)	23 (9 + 14)
Total no.	21 (17 + 4)		7 (4 + 3)		31 (11 + 20)	

We followed *LEC1* and *DR5v2* reporter expression in structures that developed into suspensorless embryos. The single‐ and few‐celled structures that developed into suspensorless embryos showed a transient decrease in *LEC1:LEC1:GFP* expression at the one‐ to two‐cell stage, followed by an increase in *LEC1:LEC1:GFP* expression at the two‐ to five‐cell stage, that is, one or two cell divisions later (Figure 1A3–A5; Figure S4A3). Exine rupture did not induce a spatial change in the *LEC1* expression pattern (Figure 1A8; Figure S4A8). After exine rupture, *LEC1* continued to be expressed in about half of the suspensorless embryos (Figure 1; Figure S4; Table 1), while the remaining suspensorless embryos lost *LEC1* expression despite continued cell division (Table 1). *LEC1* expression was maintained in embryogenic calli that developed from suspensorless embryos after exine rupture (Table 1).

The *DR5v2* reporter was also expressed at the start of the tracking period in single‐ and few‐celled embryos that developed into suspensorless embryos (Figure 2A1; Figure S5A1; Table 2). *DR5v2* expression decreased transiently in two‐celled embryos (Figure 2A1–A3; Figure S5A1,A2) and then increased during the four‐ to five‐cell stage (Figure 2A4; Figure S5A3). Resumption of *DR5v2* expression after the two‐cell stage occurred earlier than was observed for *DR5:GFP* expression, which only became visible again at the 15‐ to 16‐cell stage of embryo development, upon stretching of the exine (Soriano et al., 2014). The earlier expression of the *DR5v2* reporter might reflect the increased sensitivity of this reporter (Liao et al., 2015). After exine rupture, *DR5v2* expression was observed throughout the embryo in about half of the suspensorless embryos (Table 1; Figure 2A5,A6). The DR5v2‐expressing structures were not tracked long enough to observe a shift in *DR5v2* expression to the basal pole of the microspore embryo (Soriano et al., 2014). *DR5v2* expression, like *LEC1* expression, was lost in some suspensorless embryos that continued to divide after exine rupture (Table 1; Figure S5A5,A6). Previously, we were unable to determine which of the four embryo development pathways is marked by *DR5:GFP* expression in one‐ and two‐celled structures (Soriano et al., 2014). The above tracking data indicate that like *LEC1* expression, *DR5v2* expression also marks the first divisions of suspensorless embryos.

In summary, our data suggest that (1) embryogenic cells that follow the suspensorless embryo pathway develop predominantly after a symmetric division of either the microspore and/or the vegetative cell of the bicellular pollen, (2) suspensorless embryos develop from structures in which exine rupture occurs relatively late in development, as shown previously, and (3) *LEC1* and *DR5v2* reporters mark the entire pathway of suspensorless embryo development, from the single cell to the globular embryo, with the exception of a transient drop in expression during the first few cell divisions.

### Suspensor embryo pathway

**Figure 3:**
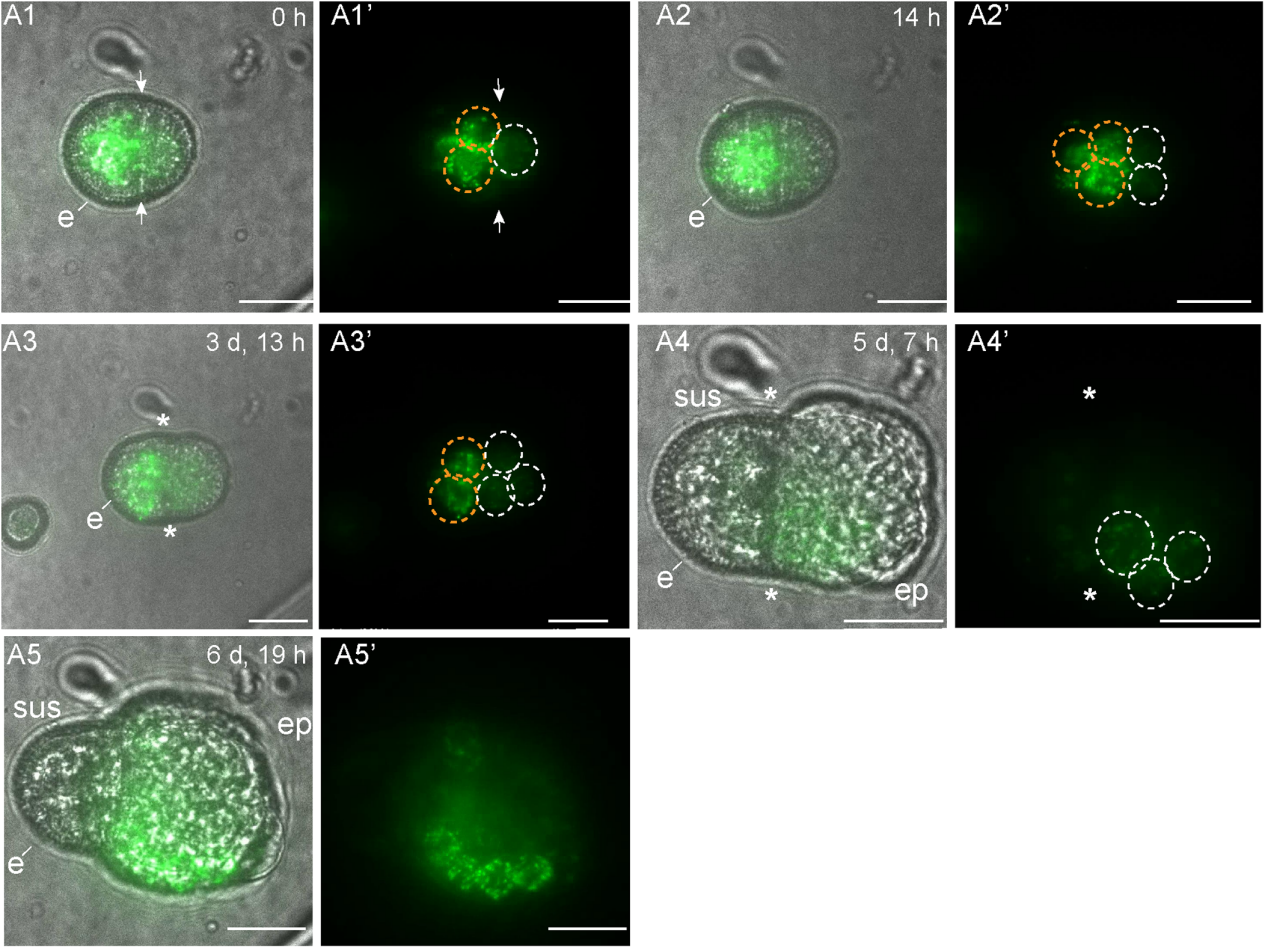
*LEC1:LEC1‐GFP* expression during suspensor‐bearing embryo development. The suspensor‐bearing embryo divided asymmetrically before the start of tracking (A1). Exine rupture started on day 3 of tracking (A3). The exine rupture plane was parallel to the first cell division plane. A multicellular structure developed with an embryo proper and a smaller suspensor that was subtended by the exine (A4, A5). Initially, relatively strong LEC1‐GFP expression was observed in the future suspensor and weaker expression in the future embryo proper (A2, A3). After exine rupture, *LEC1:LEC1‐GFP* expression gradually increased in the embryo proper, while *LEC1:LEC1‐GFP* expression in the suspensor gradually decreased (A4, A5). For each timepoint, a transmission image is shown next to the fluorescence image. The green signal corresponds to GFP fluorescence. White arrows indicate the first embryogenic cell division plane; white dashed circles indicate apical nuclear LEC1 expression; orange dashed circles indicate basal nuclear LEC1 expression; e, exine; *, site of exine rupture; sus, suspensor; ep, embryo proper. The videos used for this figure can be found in Supporting Information. Scale bar = 10 μm.

**Figure 4:**
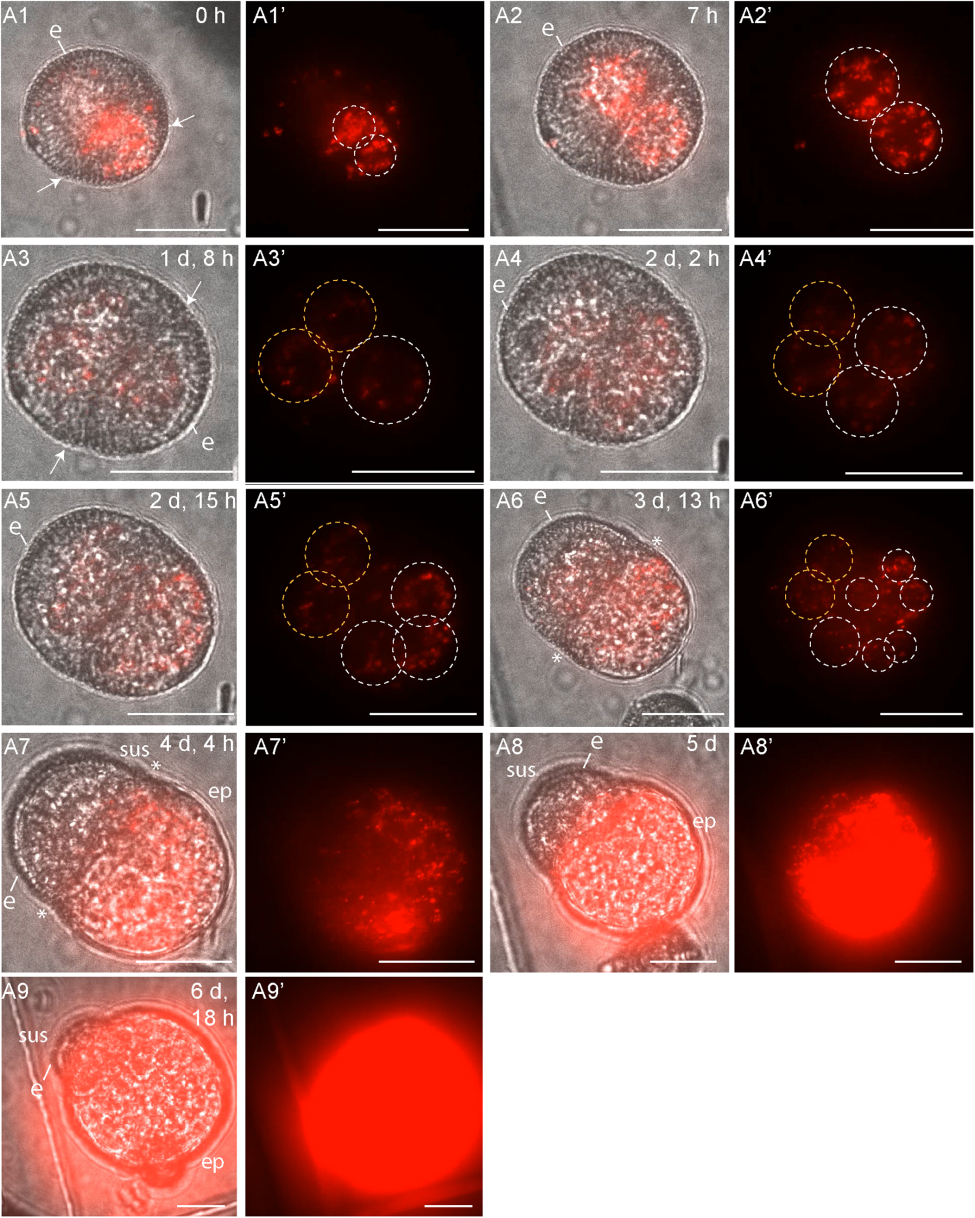
*DR5v2:ntdTomato* expression during suspensor embryo development. Suspensor embryos developed after an initial asymmetric division (A1, A3), as inferred from the cell division plane and position of the two nuclei within the dividing structure. DR5v2:ntdTomato expression was observed in the cells that contributed to both the future embryo proper and suspensor (A1, A2). In A4–A6, the nuclei in the two‐celled suspensor are indicated with orange dashed circles and the future embryo proper with white dashed circles. Exine rupture started on day 3 of tracking (A3) and occurred parallel to the first embryogenic division plane (A3). After exine rupture, DR5v2:ntdTomato expression was only observed in the embryo proper (A5–A9). For each timepoint, a transmission image is shown next to the fluorescence image with the same label. The relatively high tdTomato fluorescence intensity of the images in panel pair A9 was reduced by approximately 10%. The red signal corresponds to tdTomato fluorescence. sus, suspensor; ep, embryo proper; e, exine; asterisk, site of exine rupture; white arrows indicate the first embryogenic cell division plane. Scale bar = 10 μm. The videos used for this figure can be found in Supporting Information.

Of the 125 tracked structures, 10 structures started development as a suspensor embryo, of which eight continued to follow this developmental pathway to the end of the tracking period (Table 1). The first embryogenic division of suspensor embryos was observed during tracking in one microspore and inferred from 6 two‐celled structures present at the start of tracking (Table 2). In these seven structures, the plane of the first embryogenic cell division was asymmetric and generated two embryogenic daughter cells with large, similarly sized nuclei (Figures 3A1 and 4A1). This cell division pattern differs from pollen mitosis I, where a larger vegetative nucleus and a smaller generative nucleus are formed (Figure S1K). As the embryogenic divisions were inferred and a generative cell reporter was not used, we could not determine whether suspensor embryos were derived from an asymmetrically divided microspore or from an asymmetrically divided vegetative cell of a bicellular pollen. The initial asymmetric division of suspensor embryos was followed by a symmetric division of both daughter cells, one of which contributed to the apical lineage and one to the basal lineage (Figures 3 and 4). Exine rupture in suspensor‐bearing embryos occurred on day 3 of tracking parallel to the division plane, between two cells or tiers of cells, such that the exine initially covered both the apical and basal cells to some extent (Figures 3A3 and 4A6; Figure S6). Later, the exine was only clearly attached to the basal cell(s) that form the suspensor (Figures 3A3 and 4A7; Figure S6A2). Exine rupture was accompanied by a morphological change in the embryo apical‐basal axis, resulting in the establishment of the embryo proper and the suspensor (Figures 3A3 and 4A7; Figure S6A3). At the end of the tracking period, suspensor embryos comprised a larger multicellular embryo proper and a smaller few‐celled suspensor. Of the remaining structures that started development as suspensor embryos (2/10), one structure arrested after only a few cell divisions, while a second structure showed extensive exine rupture and developed into a loose embryogenic callus (Table 1).

Initially, the *LEC1* reporter was expressed in all cells of the future suspensor embryo (Table 2; Figure 3A; Figure S6A). Prior to or at visible exine rupture, *LEC1* expression became relatively stronger in the cell(s) that developed into the suspensor and weaker in the cell(s) that developed into the embryo proper (Figure 3A2,A3; Figure S6A). After exine rupture, the *LEC1* reporter expression was consistently observed in the embryo proper, while expression in the suspensor was variable (Figure 3A4,A5; Figure S6A4). The few suspensor embryos that stopped dividing (*n* = 1) or developed into an embryogenic callus (*n* = 1) continued to express the *LEC1* reporter (Table 1).

We also followed *DR5v2* expression in two suspensor‐bearing embryos throughout the tracking period. Initially, both the future apical and basal cells of these suspensor embryos showed *DR5v2* expression (Figure 4A1–A6), but shortly after exine rupture, *DR5v2* expression became restricted to the apical (future embryo proper) cells (Figure 4A7–A9).

Static imaging and time‐lapse imaging studies from day 5 of culture showed that *LEC1* and *DR5* expressions in haploid suspensor embryos and zygotic embryos are similar, with *LEC1* expression in both the embryo proper and suspensor and *DR5v2* expression specifically in the embryo proper (Corral‐Martínez et al., 2020; Li et al., 2014) (Figure S1). Here, we show that the *DR5v2* and *LEC1* reporters are also expressed earlier in suspensor embryo development, in the single‐ and few‐celled stages, and that their subsequent expression patterns change upon exine rupture. After exine rupture, *LEC1* expression increased in the embryo proper, while *DR5v2* expression shifted from the basal suspensor to the embryo proper cells. These data suggest that exine rupture is not only a signal for apical‐basal polarity establishment during *in vitro* suspensor embryo development but is also accompanied by dynamic changes in gene expression. However, these data should be interpreted with caution: DH12075 microspores rarely develop into suspensor embryos, and thus, we were only able to track a low number of cells that developed into suspensor embryos.

### Embryogenic callus pathway

**Figure 5:**
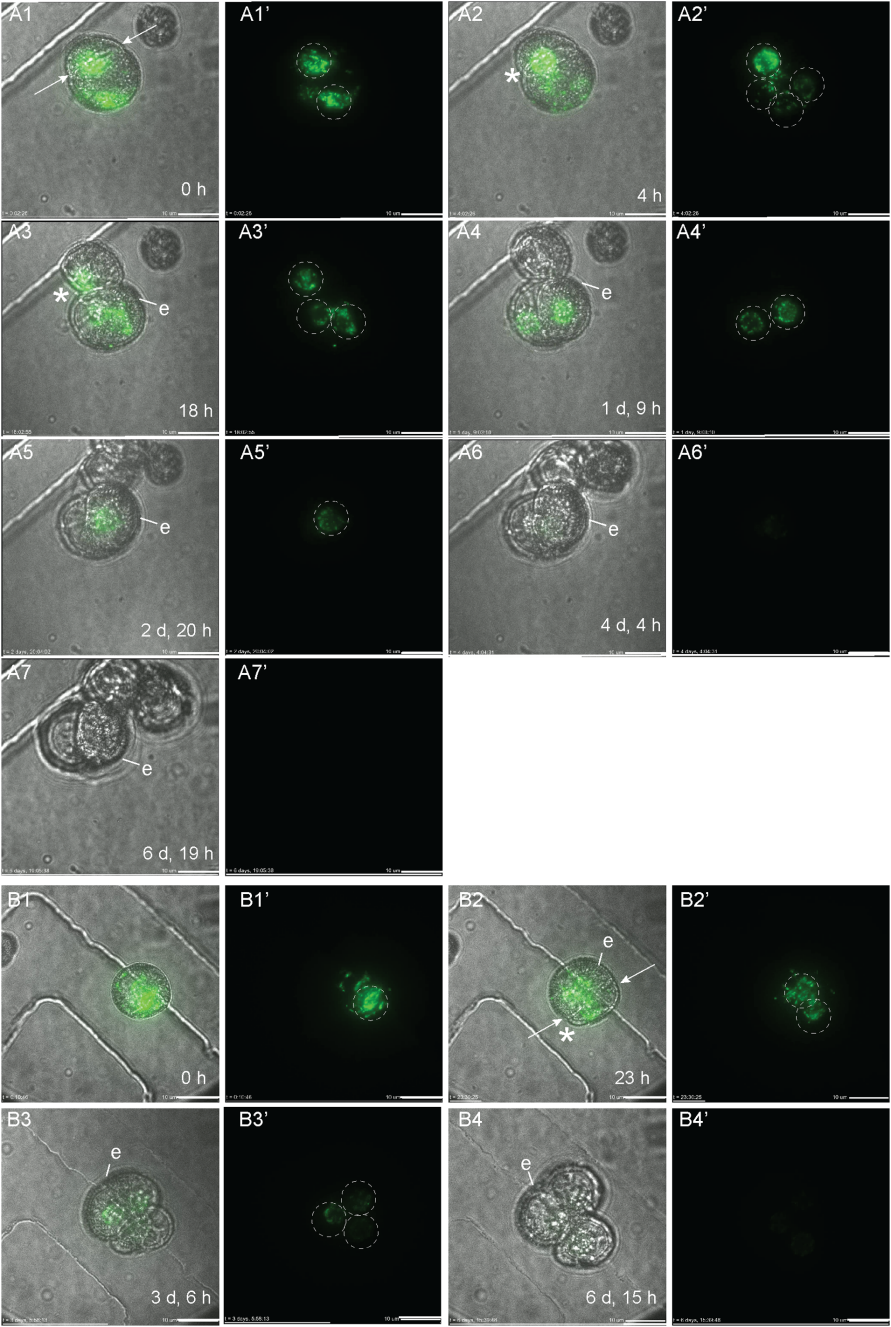
*LEC1:LEC1‐GFP* expression during embryogenic callus development. (A1–A7) Loose embryogenic callus development. Loose embryogenic callus development started with an asymmetric cell division that resulted in two large and equal‐sized nuclei (A1). Complete exine rupture occurred 4 h after the start of tracking (A2). The embryogenic structure lost *LEC1:LEC1‐GFP* expression in the uppermost cell that was no longer attached to the exine, while cells partially sheathed by the exine retained *LEC1:LEC1‐GFP* expression for a longer period of time (A3–A5). (B1–B4) Compact embryogenic callus development. The first embryogenic division was asymmetric (B2). The structure showed early (23 h) and partial exine rupture (B2). The partial exine rupture was followed by the gradual immediate loss of *LEC1:LEC1‐GFP* expression in the lower cell (B3) and gradually loss of *LEC1:LEC1‐GFP* expression in the upper two cells. For each timepoint, a transmission image is shown next to the fluorescence image labeled with the same letter. The green signal corresponds to GFP fluorescence. White arrows indicate the first embryogenic cell division plane; white dashed circle indicates nuclear *LEC1:LEC1‐GFP* expression; e, exine. The videos used for this figure can be found in Scale bar = 10 μm. Supporting Information.

About one third of the tracked cells/structures (46/125) developed directly (*ab initio*) into embryogenic callus (Table 1). The vast majority of calli for which the first embryogenic cell division could be determined (28/31) developed after an asymmetric division (Table 2). Asymmetric divisions were observed in microspores (Figures 5B1,B2 and 6A1,A2) or inferred from asymmetrically divided two‐cell structures present at the start of tracking (Figures 5A1 and 6B1, Figure S7B1). As with suspensor embryos, most asymmetric divisions generated cells with two similarly sized nuclei. However, for two structures, we observed *LEC1‐*expressing structures with two differently sized nuclei (Figure S7B1), resembling the larger vegetative and smaller generative nuclei of bicellular pollen (Figure S1K). The initial asymmetric division of embryogenic calli was followed by symmetric division of the larger vegetative‐like nucleus (Figure S7B2). Similar observations were reported previously using static imaging of the *GRP* embryo identity reporter, where up to two *LEC1*‐expressing generative‐like cells were observed (Soriano et al., 2014).

**Figure 6:**
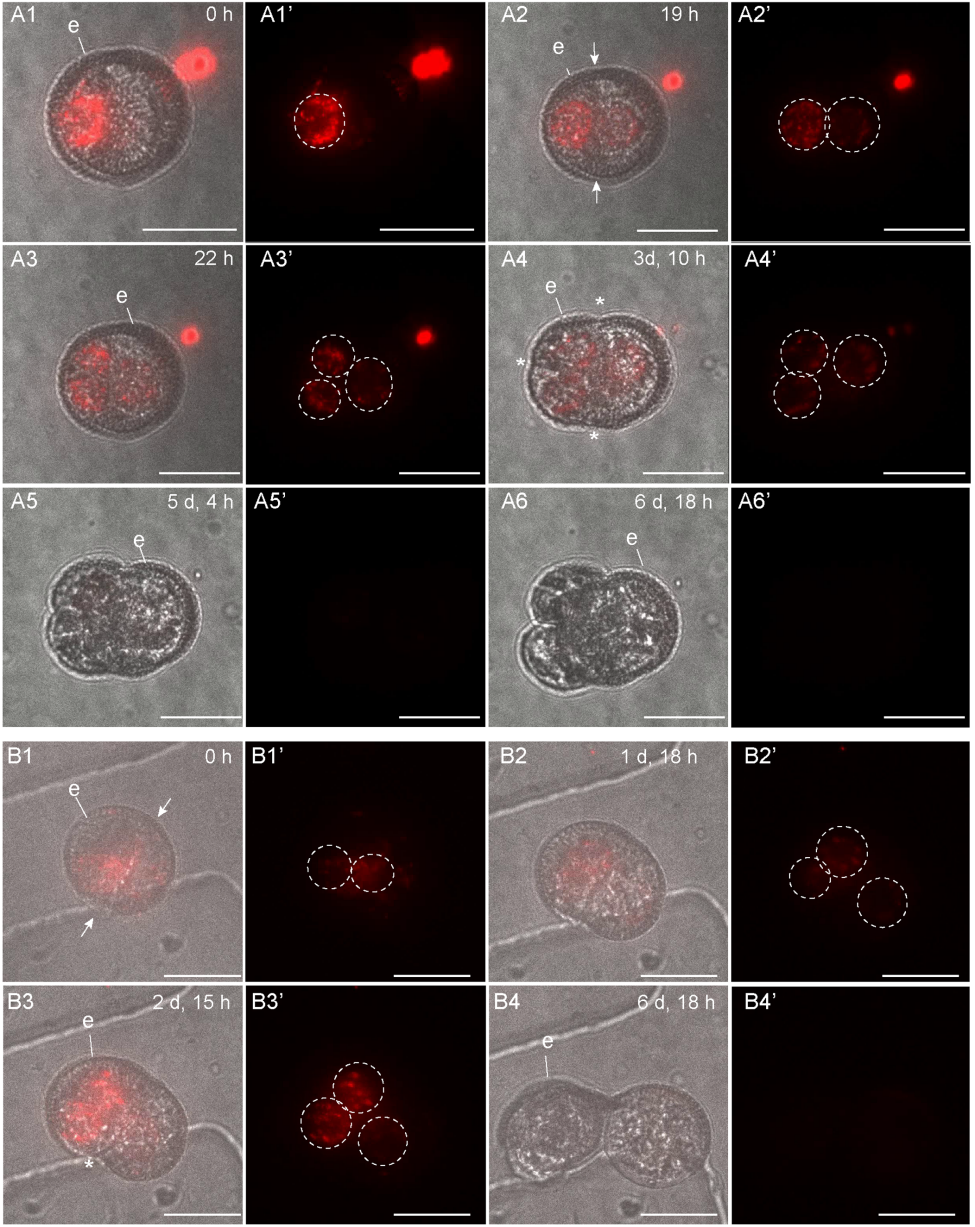
*DR5v2:ntdTomato* expression during embryogenic callus development. (A1–A6) show compact embryogenic callus development. Compact and loose callus development begins with an asymmetric embryogenic division (A2, B1). The first embryogenic cell division was asymmetric (A1, A2). Exine rupture was initiated on day 3 of tracking (A4). DR5v2 expression was lost in both callus structures, including cells that remain attached to the exine (A5, A6). (B1–B4) show loose embryogenic callus development. The first embryogenic division is asymmetric (B1). Exine rupture was initiated on day 2 of tracking (B3). DR5v2:ntdTomato expression was lost in all cells, even those covered by the exine (B4). For each timepoint, a transmission image is shown next to the fluorescence image with the same label. The red signal corresponds to ntdTomato fluorescence. White arrows indicate the first embryogenic cell division plane; white dashed circles indicate nuclear DR5v2 expression; e, exine; asterisk, site of exine rupture. The videos used for this figure can be found in Scale bar = 10 μm. Supporting Information.

Embryogenic callus development was characterized by early exine rupture, between days 1 and 3 of tracking, at the two‐ to six‐cell stage (Figures 5A2,B2 and 6A4,B3; Figure S7A3,B3). Compact callus showed partial exine rupture and loosely attached cells (Figures 5B4 and 6A6; Figure S7A4), while loose callus structures showed complete exine rupture and extensive loss of cell adhesion (Figures 5A6 and 6B4; Figure S7B5). Unlike the embryogenic calli that developed from suspensorless or suspensor embryos, embryogenic calli structures that developed *ab initio* stopped dividing immediately or shortly after exine rupture (Table 1).

*LEC1* expression was observed from the start of culture in single‐ and few‐celled structures that developed into embryogenic calli (Table 1). Individual callus cells that were partially sheathed by the exine gradually lost *LEC1* expression, while calli cells that were no longer surrounded by the exine showed rapid loss of *LEC1* expression (Figure 5A4–A7,B3,B4; Figure S7A3,A4,B4,B5). Single and multicellular structures that developed into embryogenic calli showed an initial *DR5v2* auxin response prior to or at exine rupture (Figure 6A3,A4,B3), which was lost soon after exine rupture in both compact calli and loose calli, even in cells that remained attached to the exine (Figure 6A5,B4).

The above data suggest that embryogenic calli, like suspensor‐bearing embryos, develop predominately from an initial asymmetric division of the microspore/vegetative cell. The few calli that originated from a symmetric first division might represent exine‐enclosed/suspensorless embryos that developed into embryogenic calli after only one cell division. Both *LEC1* and *DR5v2* are expressed in embryogenic calli, but their expression is lost after exine rupture. *LEC1* expression is initially maintained in cells that remain partially attached to the exine, while *DR5v2* expression is rapidly lost in all cells after exine rupture.

## DISCUSSION

**Figure 7:**
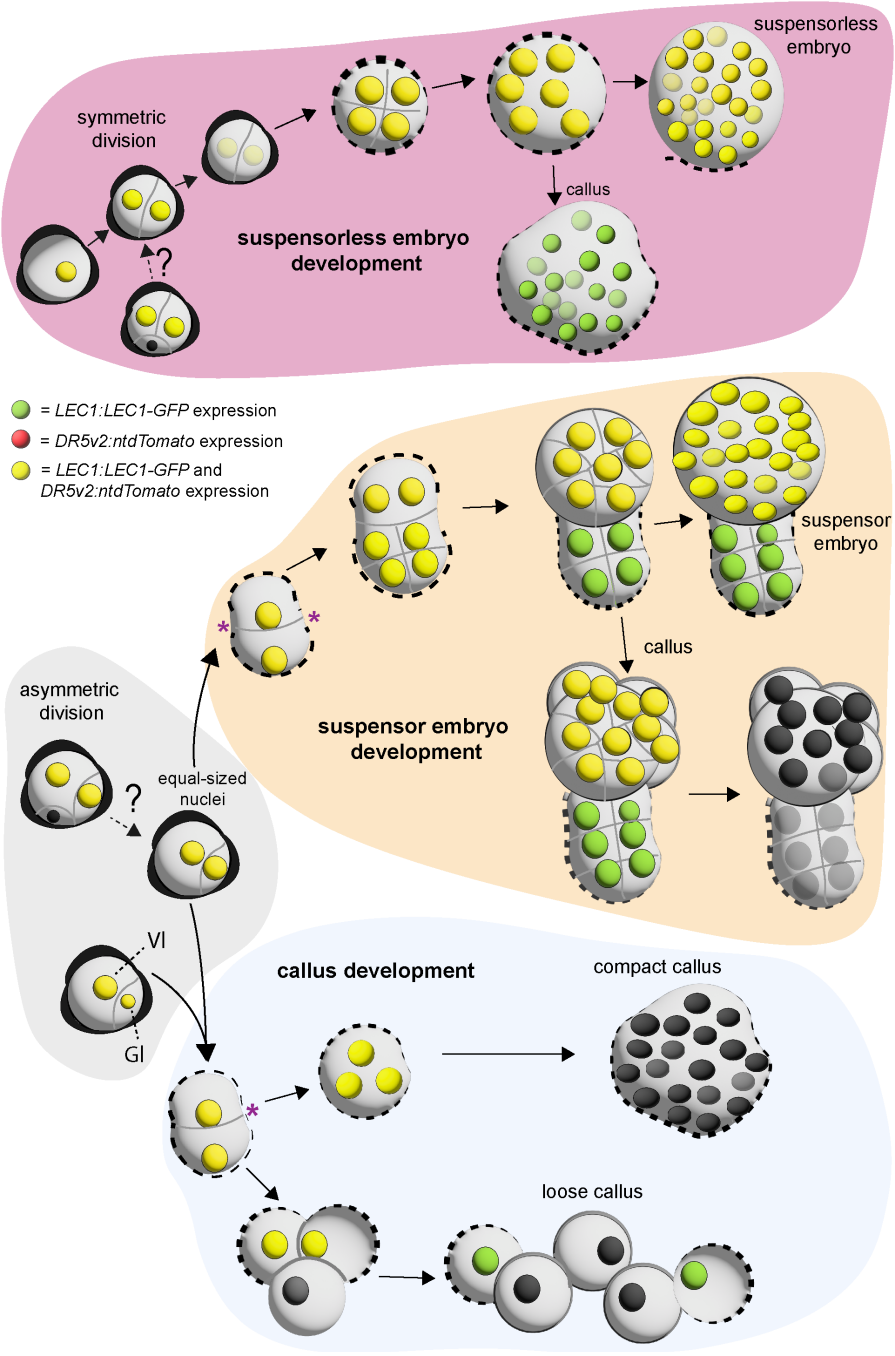
Schematic model of the main developmental pathways of embryogenic cells during *Brassica napus* microspore embryogenesis. Four different types of embryogenic structures can be recognized in culture: suspensorless embryos, suspensor‐bearing embryos, and compact and loose embryogenic callus. The four developmental routes can be distinguished based on the orientation of their first embryogenic division, the timing and extent of exine rupture, and the expression pattern of the *LEC1:LEC1‐GFP* (green) and *DR5v2:ntdTomato* (red) reporters. Suspensorless embryo development is initiated by a symmetric division of either the microspore or the vegetative cell of the bicellular pollen, followed by continued divisions of the two daughter cells inside the exine. Exine rupture occurs relatively late in the tracking period. A proportion of these exine‐enclosed structures develop into embryogenic callus. Suspensor‐bearing embryos and embryogenic callus are derived from an initial asymmetric division of either the microspore or the vegetative cell of the bicellular pollen. In embryogenic callus, the asymmetric division produces two cells that have two equal‐sized nuclei or nuclei that differ in size, resembling a smaller generative‐like (Gl) and a larger vegetative‐like (Vl) cell. Only the cells with vegetative‐like nuclei develop into the embryogenic callus. Compact and loose embryogenic callus show relatively early exine rupture, with compact callus showing partial rupture and loose callus showing complete rupture of the exine. These calli undergo limited cell divisions. In suspensor embryos, an asymmetric cell division produces two differently sized cells with two equal‐sized nuclei. Suspensor‐bearing embryos develop a larger apical multicellular embryo proper and a smaller basal few‐celled suspensor that is usually subtended by the original pollen wall. Suspensor‐bearing embryos can also develop into callus. The exine is indicated by a dotted line around the embryogenic structure.

Our earlier static imaging studies showed that embryo identity markers (*GRP* and *LEC1*) and a *DR5*‐based auxin response marker (*DR5rev:GFP*) are expressed in the single‐ to two‐celled structures that are found on days 2–3 of microspore culture (Li et al., 2014; Soriano et al., 2014). The subsequent developmental pathways marked by *LEC1* and *DR5* expression could not be determined in these few‐celled structures, as suspensorless embryos, suspensor embryos, and embryogenic callus can only be morphologically distinguished from each other later, around day 5 of culture (Soriano et al., 2014). Time‐lapse imaging has been used to follow the early development of dividing cells in microspore cultures of wheat (Indrianto et al., 2001) and barley (Daghma et al., 2014; Maraschin et al., 2005, 2008). These studies documented the cell division patterns associated with the transition from gametophyte to sporophyte development. However, the lack of embryo identity reporters made it impossible to define the initial identity and fate of the multicellular structures that develop in these cultures. Previously, we used time‐lapse imaging to follow the development of embryogenic structures that express the *LEC1:LEC1‐GFP* embryo identity reporter starting on day 5 of culture (Corral‐Martínez et al., 2020). These embryogenic structures could already be morphologically identified as suspensorless embryos, suspensor embryos, and embryogenic calli at the start of tracking. Using this approach, we characterized the viability and final developmental fate of these multicellular embryogenic structures and showed that embryogenic calli develop *ab initio* or from suspensor‐bearing or suspensorless embryos. Here, we followed the development of single‐ to few‐celled structures that expressed the *LEC1:LEC1‐GFP* embryo identity reporter or the *DR5v2:ntdTomato* auxin response reporter. Combining time‐lapse imaging with earlier imaging of embryo identity and auxin response reporters at the single‐ to few‐cell stage of microspore embryogenesis allowed us to identify the developmentally plastic trajectories that generate embryogenic structures, the role of the first embryogenic division plane in defining cell fate, and the role of mechanical changes in the pollen wall in orchestrating gene expression patterns and cell identity (Figure 7). The many developmental pathways that generate embryogenic structures in *B. napus* microspore cultures stand in stark contrast to the regular cell division patterns that generate *B. napus* zygotic embryos (Tykarska, 1976, 1979), suggesting that embryo development pathways are inherently plastic and context‐dependent.

### The relationship between cell division plane, exine rupture, and cell fate

Haploid embryo development in *B. napus* microspore culture can be initiated from cells that divide symmetrically or asymmetrically (Figure 7). Earlier static imaging studies in *B. napus* microspore cultures suggested that embryos develop after an initial symmetric division of the microspore or the vegetative cell (Telmer et al., 1995; Zaki & Dickinson, 1990). Here, we show that embryogenic microspores also divide asymmetrically. We observed asymmetrically divided cells at the start of tracking, but due to the reporters used, we could not determine whether the microspore or the vegetative cell of a bicellular pollen divided asymmetrically. Asymmetric cell divisions leading to the formation of embryogenic structures might have been overlooked in these earlier studies for three reasons: (1) asymmetrically divided structures were still considered to be pollen; (2) what we now refer to as embryogenic calli were previously considered to be non‐embryogenic structures (Ilić‐Grubor et al., 1998; Telmer et al., 1995); and (3) in *B. napus* cv Topas DH4079, the model genotype used at that time, the first embryogenic division of suspensor embryos takes place much later than that of suspensorless embryos (Joosen et al., 2007).

Regardless of the initial division plane, *B. napus* embryogenic structures always developed from either the microspore or the vegetative cell of a bicellular stage pollen (Figure 7). Occasionally, we observed one or more generative‐like cells that expressed embryo identity markers (Figure S7; Soriano et al., 2014), but these cells did not contribute further to embryo development. Development of microspore embryos from either the generative cell or both the generative and microspore/vegetative cell have been described, but in most species, the microspore/vegetative cell develops into the embryo (Raghavan, 1997; Sunderland, 1973). More often, generative‐like cells divide slowly to produce a few cells or a syncytium of free nuclei that do not contribute to embryo development (Daghma et al., 2014; Raghavan, 1997). Intriguingly, in *Hyoscyamus niger*, the generative cell is thought to form the embryo proper and the vegetative the suspensor (Raghavan, 1976, 1978). Thus, depending on the context, the different cells of the immature pollen grain all have the developmental potential to undergo additional cell divisions and/or develop into an embryo/embryogenic structures.

The orientation of the first embryogenic cell division is an early morphological predictor of developmental fate (Table 2; Figure 7). We observed that symmetric division of the microspore or vegetative cell leads predominantly to suspensorless embryo development, while asymmetric divisions lead predominantly to suspensor embryo and embryogenic callus formation. Symmetric division of the *B. napus* microspore or vegetative cell in microspore culture is associated with the formation of a preprophase band, which is absent during pollen mitosis (Simmonds & Keller, 1999). The preprophase band is a ring of cortical microtubules circumscribing the nucleus that predicts the position and orientation of the future cell division plane in dividing somatic cells (Rasmussen & Bellinger, 2018). Treating *B. napus* microspores with microtubule depolymerizing agents enhances microspore embryo yields, which is attributed to the increase in symmetric cell divisions (Zaki & Dickinson, 1990; Zhao et al., 1996). We observed that suspensor embryos and calli also develop after an asymmetric division of an embryogenic microspore or vegetative cell, indicating that symmetric cell division is not a prerequisite for establishing embryo identity. Colchicine does not induce microtubule destabilization in bicellular pollen (Zhao & Simmonds, 1995); thus, the orientation of the division plane induced after the abiotic stress treatment and the subsequent development of embryogenic structures might be context‐dependent: Relatively younger gametophytic cells with microtubule configurations that are sensitive to abiotic stress/colchicine‐induced depolymerization divide symmetrically, while relatively older gametophytic cells with microtubule configurations that are not sensitive to abiotic stress/colchicine‐induced depolymerization divide asymmetrically. It is not clear why symmetric divisions generate predominantly suspensorless embryos and why asymmetric divisions generate predominantly suspensor embryos and embryogenic calli, but stage‐specific gametophytic factors like cell wall composition and polarity factors might determine the establishment of these different embryogenic pathways.

Our data suggest that the first embryogenic cell division plane is a key determinant of cell fate, with suspensorless embryos developing predominantly after an initial symmetric cell division and suspensor embryos/embryogenic calli predominantly after an initial asymmetric division (Table 2; Figure 7). However, Tang et al. (2013) have shown that suspensor embryos can also develop after either a symmetric or asymmetric division when the exine of *B. napus* microspores is artificially broken prior to culture but remains attached to most of the cell surface. This observation suggests that cell fate and division symmetry can also be uncoupled and highlights the context‐dependent developmental plasticity of microspore embryogenesis. If the cell division plane *per se* does not determine cell fate in microspore culture, then what does? In most microspores with exines that were artificially ruptured prior to culture, the position of the first division plane is defined by the site of exine rupture, which occurs parallel to the division plane (Tang et al., 2013), as was also observed here (Figures 3 and 4). Ultrastructural analyses of microspores with exines that were artificially ruptured prior to culture showed that cellular polarity is apparent immediately after exine rupture and before the first cell division: one cell pole is rich in organelles and presumed to become the embryo proper, while the other cell pole is vacuolate and organelle‐poor and was presumed to develop into the suspensor. Thus, exine stretching or rupture might provide mechanical and/or chemical cues that induce changes in cell polarity without the requirement for an asymmetric cell division.

Exine rupture in microspore embryo cultures, as performed in this study, must be driven by intrinsic factors. During suspensorless embryo development, the exine stretches in response to the increase in cell number (Figures 1 and 2), eventually leading to exine rupture at the globular stage (Soriano et al., 2014). By contrast, during suspensor embryo development (Figures 3 and 4) and embryogenic callus development (Figures 5 and 6), the exine ruptures at the few‐celled stage. This suggests that different mechanisms drive exine rupture in the different types of embryogenic structures. Suspensor embryos, and more so embryogenic calli, have greatly enlarged cells, and thicker and less flexible cell walls compared to suspensorless embryos (Figure S1; Camacho‐Fernández et al., 2021). Suspensor embryos and embryogenic calli might experience very high turgor pressure that is initially counteracted by thickening and stiffening of cell walls; however, when the turgor pressure exceeds the tensile strength of the exine, the exine ruptures, allowing the no longer mechanically restrained cells to expand excessively and detach. The initial embryogenic cell division plane and the timing of exine rupture appear to be intimately connected events, but whether one is causal for the other is not known. Future studies aimed at charting the mechanobiology landscape of the different types of embryogenic structures before and after cell division and exine rupture will provide insight into why and how these structures develop.

### Dynamic *LEC1* and *DR5v2* expression patterns during microspore embryogenesis

We followed the development of single‐ and few‐celled structures marked by the expression of either the *LEC1* embryo identity reporter or the *DR5v2* auxin output reporter. The *LEC1* transcription factor gene regulates zygotic embryo and endosperm expression (Jo et al., 2019), and its expression marks embryo identity in different *in vitro* embryo culture systems (Horstman et al., 2017; Kadokura et al., 2018; Li et al., 2014), while *DR5*‐based reporters mark transcriptional activity induced by auxin, but not necessarily auxin levels *per se* (Jedličková et al., 2022). In *B. napus* microspore embryo cultures, both the *LEC1* and *DR5v2* reporters specifically mark the development of embryogenic structures at the single‐ to few‐cell stage, but with different temporal and spatial expression patterns in the different types of embryogenic structures that develop subsequently (Figure 7; Li et al., 2014; Soriano et al., 2014).

The *LEC1* and *DR5v2* reporters showed a transient decrease in expression in few‐celled suspensorless embryos (Figures 1 and 2). This dynamic expression pattern might reflect a dilution of initial *LEC1‐GFP* and *ntdTomato* mRNA or protein levels, but it might also mark a key event that takes place during suspensorless embryo development. However, this transient reduction in *DR5v2* and *LEC1*‐driven reporter expression is not followed by any obvious morphological changes. One possibility is that suspensorless embryo fate is not completely fixed at the few‐cell stage and needs to be reinforced by an undefined signal to promote further development. Exine‐enclosed structures that stop dividing after a few cell divisions continue to express the *LEC1* reporter until the end of the tracking period, but do not express the *DR5v2* reporter (Table 1). These structures might stop dividing at a developmental state that precedes this critical signal, resulting in loss of *DR5v2* expression but not *LEC1* expression. Auxin response minima or auxin depletion has been associated with several developmental events including stomatal cell patterning, axillary meristem and lateral root initiation, leaf polarity, and root cell differentiation (Di Mambro et al., 2017; Dubrovsky et al., 2011; Le et al., 2014; Qi et al., 2014; Wang et al., 2014). In these systems, auxin minima occur in specific cell types or zones within a larger tissue, while the transient reduction in *DR5v2* expression observed during suspensorless embryo development is observed in all cells of the few‐celled embryo. It is, therefore, unlikely that a transiently reduced auxin response at the few‐cell stage drives the development of a new haploid embryo cell or tissue type that in turn drives further development of the suspensorless embryo. Rather, the reduced auxin response and decreased *LEC1* reporter expression in few‐celled suspensorless embryos might signal a critical cell fate decision point that is resolved in a self‐organizing fashion. This critical cell fate decision point might never occur in suspensor embryos and embryogenic callus due to different signaling events that are set in motion by the relatively earlier exine stretching in these structures. Single‐cell transcriptome and live‐imaging studies could be used to define the nature of this dynamic expression pattern.

Prior to exine rupture, all suspensor‐bearing embryo cells express the *LEC1* and *DR5v2* reporters (Figures 3, 4, and 7). After exine rupture, the future basal/suspensor and apical/embryo proper become morphologically recognizable, and the LEC1 and DR5 expression patterns change: *LEC1* expression is induced in the apical/embryo proper cells and (variably) maintained in the suspensor cells, while *DR5v2* expression is restricted to the embryo proper (Figures 3, 4, and 7). This pattern differs from the abrupt loss of *DR5v2* and *LEC1* reporter expression that is observed after exine rupture in embryogenic calli (Figures 5, 6, and 7). Two factors might contribute to the maintenance of *DR5v2* and *LEC1* reporter expression in suspensor embryos: (1) the initial presence of the exine on apical and basal pole cells (although in different amounts) and (2) the better intercellular adhesion (and thus intercellular communication) in suspensor embryos compared to embryogenic calli (Camacho‐Fernández et al., 2021; Corral‐Martínez et al., 2020).

The switch to an apically dominated *DR5v2* reporter expression pattern suggests that exine rupture induces changes in auxin biosynthesis, transport, or signaling. The change in cell polarity that accompanies exine rupture (Tang et al., 2013) might stimulate *PIN* or *AUX/LAX* gene expression resulting in shootward auxin transport. The PIN7 auxin efflux carrier is expressed in the apical cell membrane of the zygotic embryo suspensor where it transports auxin to the embryo proper, resulting in a *DR5* maximum (Friml et al., 2003; Robert et al., 2013). *PIN7:PIN7‐GFP* expression in the apical membrane of the suspensor of microspore embryos has also been observed, but these embryos were more developmentally advanced than the few‐celled structures observed here (Soriano et al., 2014). Time‐lapse imaging of *PIN7:PIN7‐GFP* lines will provide information on whether *PIN7* expression is also induced in few‐celled suspensor embryos upon exine rupture. Dynamic changes in expression of the *LEC1* embryo identity gene caused by exine rupture are more difficult to explain. One possibility is that changes in *LEC1* expression reflect a transcriptional response to changes in auxin levels, as observed in auxin‐induced somatic embryo cultures (Ledwon & Gaj, 2011).

Embryogenic calli that developed *ab initio* lost both *LEC1* and *DR5v2* expressions after exine rupture, but with different dynamics (Figures 5 and 6). *DR5v2* expression was lost after exine rupture, regardless of whether the cells remained attached to the exine, while *LEC1* expression was initially only lost in cells that had detached from the exine. Cells that are covered by exine have more connecting fibers and higher levels of highly methyl‐esterified pectin in their cell walls than cells that have detached from the exine (Camacho‐Fernández et al., 2021; Corral‐Martínez et al., 2013). This raises the possibility that in addition to maintaining cell adhesion and mechanical pressure, pectin or other cell wall components that connect the embryogenic callus cells to the pollen wall might be needed to maintain cell fate (Shin et al., 2021).

## EXPERIMENTAL PROCEDURES

### Plant material and culture

The *B. napus* DH12075 genotype was used for all experiments. DH12075 is a spring‐type doubled‐haploid derived from a cross between Westar and Cresor (G. Seguin Schwartz and G. Rakow, Agriculture and Agrifood Canada Saskatoon, Canada). The plant growth conditions and microspore culture procedures were performed as described previously (Corral‐Martínez et al., 2013; Custers, 2003). Prior to time‐lapse imaging, isolated microspores and early stage bicellular pollen (4 × 10^4^/mL) from 10°C/5°C (16 h/8 h)‐grown plants were cultured at 33°C with 0.05 μM TSA for 24 h (HS) in 15‐mL plastic tubes (Greiner; 9 mL NLN‐13 medium/tube), after which the TSA was removed by centrifugation (3 min at 130*g*) in a cooled centrifuge and replaced with fresh liquid NLN‐13 medium. Trichostatin A (TSA) (Sigma) was dissolved in DMSO.

The *LEC1:LEC1‐GFP* reporter line was described previously (Li et al., 2014). The *DR5v2:ntdTomato* reporter construct (*DR5v2*; Liao et al., 2015) was transformed to *Agrobacterium tumefaciens* strain C58C1 pMP90 and then to DH12075 as described in Moloney et al. (1989). All reporter lines showed wild‐type phenotypes and embryo yields.

### Microscopy

DAPI staining (4′,6‐diamidino‐2‐phenylindole; 1.25 μg mL^−1^) of pollen and embryogenic structures (Custers, 2003) was used to determine the effect of embedding and time‐lapse imaging on the progression of microspore embryogenesis. Non‐immobilized cultures were stained with DAPI after removal from the Petri dish and centrifugation, while agarose‐immobilized cultures were stained directly in the Petri dish. DAPI was visualized with a Zeiss Axioskop epifluorescence microscope (excitation wavelength, 400 nm; emission wavelength, 420 nm).

For confocal laser scanning microscopy imaging, eGFP fluorescence in the *LEC1:LEC1‐GFP* reporter line was observed with a Leica DM5500 Q confocal laser scanning microscope. eGFP was excited with an argon laser line at 488 nm and detected with a 411‐ to 544‐nm emission filter. tdTomato was imaged using confocal laser scanning microscopy (Leica DM5500 Q). tdTomato was excited at 534 nm and detected with a 555‐ to 688‐nm emission filter. Cell walls (Figure S1) were counterstained for 30 min in 0.1% (v/v) SCRI Renaissance 2200 (Musielak et al., 2015). DAPI (4′,6‐diamidino‐2‐phenylindole) staining of nuclei (Figure S1) was performed as previously described (Custers, 2003). SCRI Renaissance 2200 and DAPI were excited with a 405‐nm laser, and emission was recorded between 415–476 and 458–487 nm, respectively. All optical slices were median filtered with Leica LAS AF software.

### Time‐lapse imaging

Microspores were immobilized in low‐temperature melting agarose after the 24 h HS + 0.05 μM TSA treatment. Prior to embedding, the culture density was adjusted to 5.0 × 10^6^ microspores mL^−1^ in NLN‐13 medium. Low melting (26–30°C) agarose (SeaPlaque, Duchefa) and double‐strength NLN‐13 medium were heated in a microwave and stored in microcentrifuge tubes in a 33°C block heater until use. Petri dishes with a 35‐mm grid (Ibidi, catalog no. 80156) were used for time‐lapse imaging. Equal volumes of the microspore culture, double‐strength NLN‐13 medium, and low‐temperature melting agarose were mixed thoroughly in a microcentrifuge tube and placed in the 33°C block heater. A 20‐μL drop of the above mixture was pipetted into the pre‐warmed Petri dish and then spread quickly to cover the bottom of the dish. The agarose was allowed to gel for 10 min by placing the Petri dish without the lid (to prevent condensation) on ice. Then, 500 μL of NLN‐13 culture medium was pipetted slowly along the inner side of the Petri dish to cover the agarose. For control non‐immobilized cultures, 24 h HS‐treated microspores were plated as above using only liquid NLN‐13 medium.

Time‐lapse imaging was performed at 21°C in a temperature‐controlled, dark room using a custom‐built multifocal two‐photon excitation microscope. GFP and ntdTomato were imaged using a 750‐nm laser beam that was split in a 10 × 10 hexagonal array of excitation beams using a diffractive optical element (DOE, custom‐made by Holo‐eye). The zeroth‐order of the diffraction pattern was blocked by a droplet of soldering tin deposited on a glass slide. A homogenous illumination plane was then created by transmitting the first‐order diffraction pattern onto a fast‐scanning mirror (Newport, FSM‐300‐1) that imparts a spiraling motion on the array of individual foci. The scanning mirror was driven with an Archimedean spiral which spiral‐scans the incident laser beams across the focal plane. The laser beams were focused using a 60X oil, 1.49 NA objective (Nikon, CFI60 Apochromat TIRF 60XC) mounted on a motorized Z‐axis positioning stage (PI, P‐726 PIFOC). The DOE produces an array of diffraction‐limited spots with a lattice spacing of 4 μm, resulting in an illumination area of approximately 40 × 40 μm. Two‐photon luminescence was collected by the same objective, then filtered with a dichroic mirror (Semrock, 700dcxr) and two short‐pass filters (Semrock, FF01‐720‐SP and FF01‐750‐SP) and focused on a 4.2‐megapixel backside‐illuminated sCMOS camera (Photometrics, Prime BSI).

The different types of embryogenic structures cannot be accurately distinguished based on morphological before day 4–5 of culture. Therefore, we randomly selected *LEC1:LEC1‐GFP* or *DR5v2*‐expressing cells/few‐celled structures in freshly immobilized cultures for tracking. These structures were tracked for 4–6 days. Images were collected every 1 or 2 h using Z‐stacks of 30 images, with a spacing of 2 μm for every structure at each time interval and attributed to different embryogenic structures afterwards.

Video compilation of the raw images was performed with in‐house software in Python that employed maximum intensity projection, where each projected pixel contains the maximum value of all pixels at the same 2D coordinate in the Z‐stack. Initial intensity scaling of each consecutive compiled image was based on the cumulative pixel intensity of the first image, which provided an accurate visualization of fluorescent change over time but often oversaturated the later frames. Optionally, for better visualization of the morphology, compiled images were individually scaled based on the 5th and 95th percentile of the cumulative pixel intensity of each image. The originals are available in the Supplemental Information.

## CONFLICT OF INTEREST STATEMENT

The authors have not declared a conflict of interest.

## Supporting information


**Figure S1.** Pollen and embryogenic structures found in microspore cultures. (A–H) Confocal laser scanning microscopy images of embryogenic structures at day 5 of culture expressing the *LEC1:LEC1‐GFP* (green fluorescence) (A–D) or *DR5v2:ntdTomato* (red fluorescence) reporters (E–H). White fluorescence, Renaissance SCR2200‐stained cell walls. (A, E) Suspensorless embryo after exine rupture; (B, F) suspensor‐bearing embryo after exine rupture; (C, G) compact callus; and (D, H) loose callus after exine rupture. (I–Q) Confocal laser scanning microscopy images of DAPI‐stained nuclei (blue) in gametophytic structures from a *DR5v2:ntdTomato* reporter line. All structures can be found at the start and during culture. No *DR5v2:ntdTomato* expression is observed. (I–O) Mid uninucleate microspore with the nucleus positioned in the middle of the cell. (J–P) Late uninucleate microspore just before pollen mitosis I, with the nucleus positioned close to the cell wall. (K–Q) Early bicellular pollen, with a larger vegetative nucleus (v) and a smaller generative nucleus (g). The exine (e) in I–Q shows autofluorescence in the light ranges used to detect DAPI and tdTomato. sus, suspensor; ep, embryo proper. Scale bars, A–H, 25 μm; I–Q, 5 μm.
**Figure S2.** Time‐lapse imaging of microspore embryo cultures does not affect the viability or developmental fate of embryogenic structures. *LEC1:LEC1‐GFP* cultures were treated with HS + 0.05 μM TSA for 24 h, after which the cultures were immobilized in agarose (immobilized) or transferred to the same type of Petri dishes (Ibidi) with liquid medium only (control) before being transferred to 25°C for further culture. (A) Immobilization system. Microspores/pollen (indicated as brown ovals) were embedded in agarose in a Petri dish with a coverslip‐like bottom, forming at most two layers of cells, and then covered with a layer of liquid medium. (B) The effect of immobilization on embryogenic structure development. The percentage of embryogenic structures formed in immobilized or non‐immobilized (control) cultures was counted 2, 4, and 8 days after transfer to Petri dishes. The data represent the average of three to four biological replicates (one Petri dish/replicate, ≥197 cultured cells counted/replicate). The error bars represent the standard error. Statistical analysis using a two‐tailed Student's *t*‐test (*P* ≤ 0.05) did not identify any statistically significant difference between the treatments per timepoint. (C) The types of embryogenic structures that develop in cultures after immobilization or without immobilization (control). The different types of embryogenic and non‐embryogenic (pollen) structures were counted 2, 4, and 8 days after transfer to Petri dishes. The different types of embryogenic structures include 2‐celled exine‐enclosed structures, 3 to ca. 12‐celled exine‐enclosed structures, globular‐shape suspensorless embryos in which the exine was ruptured, suspensorless embryos with ruptured exine, and loose and compact callus with ruptured exines. Three to four biological replicates were performed for each treatment (one Petri dish/biological replicate, ≥197 cells/replicate). (D) The effect of cell tracking with TPEFM on embryo development. The percentage of embryogenic and pollen structures was compared on day 8 in immobilized cultures and in immobilized and tracked cultures, 8 days after transfer to Petri dishes. Four biological replicates were performed for each treatment (one Petri dish/replicate, ≥291 cells/replicate). The error bars represent the standard error. Statistical analysis using two‐tailed Student's *t*‐test (*P* ≤ 0.05) did not identify any statistically significant difference between the different comparisons.
**Figure S3.** Arrested cell division in exine‐enclosed structures. A1–A4 show an exine‐enclosed structure that stopped dividing after a few cell divisions but continued to express the *LEC1:LEC1‐GFP* reporter in some of the cells. B1–B4 show an exine‐enclosed *DR5v2:ntdTomato*‐expressing structure that stopped dividing and lost *DR5v2:ntdTomato* expression during the tracking period. For each timepoint, a transmission image is shown next to the fluorescence image. The green signal corresponds to GFP fluorescence, and the red signal corresponds to ntdTomato fluorescence. White arrows indicate the first embryogenic cell division plane; white dashed circles indicate nuclear expression of *LEC1:LEC1‐GFP* or *DR5v2:ntdTomato*; e, exine; asterisk (*), site of exine rupture. The videos used for this figure can be found in Scale bar = 10 μm Supporting Information.
**Figure S4.** *LEC1:LEC1‐GFP* expression in a suspensorless embryo. The single‐cell microspore (A1) divided symmetrically (A3). A transient reduction in *LEC1:LEC1‐GFP* expression was observed at the two‐cell stage (A3). The exine started to rupture on day 6 of tracking (A6). A transmission image is shown next to the fluorescence image for each timepoint. The green signal corresponds to GFP fluorescence. All images were autoscaled to reduce the fluorescence intensity. White arrows indicate the first embryogenic division plane; white dashed circles indicate nuclear *LEC1* expression; e, exine; *, site of exine rupture. The videos used for this figure can be found in Scale bar = 10 μm. Supporting Information.
**Figure S5.** DR5v2:ntdTomato expression in a suspensorless embryo. The suspensorless embryo developed after an initial symmetric division (A1). DR5v2 expression decreased after the two‐celled stage (A2) and then increased at the four‐cell stage (A3). Exine rupture started 5 days after the start of tracking (A5). This suspensorless embryo started to lose the DR5v2 expression at the multicellular embryo stage (A5–A6), possibly marking an imminent switch to embryogenic callus development as described in Corral‐Martínez et al. (2020). For each timepoint, a transmission image is shown next to the fluorescence image. The red signal corresponds to ntdTomato fluorescence. White arrows indicate the first embryogenic cell division plane; white dashed circles indicate DR5v2 nuclear expression; e, exine; asterisk (*), site of exine rupture. The videos used for this figure can be found in Supporting Information. Scale bar = 10 μm.
**Figure S6.** LEC1:LEC1‐GFP expression in a suspensor embryo. A multicellular structure was observed at the start of tracking (A1). The exine started to rupture on day 3 of tracking (A2). The exine ruptured parallel to the cell division plane (A2). The structure developed into a multicellular embryo proper with a smaller few‐celled suspensor that was subtended by the exine (A3–A4). Initially, a stronger LEC1:LEC1‐GFP expression was observed in the future suspensor than in the future embryo proper (A2). After exine rupture, the LEC1:LEC1‐GFP reporter gradually became more highly expressed in the embryo proper than in the suspensor (A4). For each timepoint, a transmission image is shown next to the fluorescence image. The green signal corresponds to GFP fluorescence. White arrows indicate the first embryogenic cell division plane; white dashed circles indicate the LEC1 nuclear expression. sus, suspensor; ep, embryo proper; e, exine; asterisk, site of exine rupture. The videos used for this figure can be found in Scale bar = 10 μm. Supporting Information.
**Figure S7.** LEC1:LEC1‐GFP expression in embryogenic calli. (A1–A4) compact callus development. The four‐celled structure showed early and partial exine rupture on day 2 of tracking (A3) that was accompanied by loss of the LEC1:LEC1‐GFP expression (A4). (B1–B5) loose embryogenic callus development. Loose callus developed after an initial asymmetric embryogenic division. (B1). The nuclei differed in size, resembling the large vegetative cell and smaller generative cell of bicellular pollen (generative‐like, Gl, vegetative‐like, Vl) (B1–B2). Only the vegetative‐like cell continued to divide (B2). Loose callus development was associated with early and extensive exine burst (B4). The newly formed daughter cell that was loosely attached to the rest of the structure lost LEC1 expression, while the cells partially sheathed by the exine retained the LEC1 expression (B4, B5). The green signal corresponds to GFP fluorescence. For each timepoint, a transmission image is shown next to the fluorescence image with the same label. White arrows indicate the first embryogenic cell division plane; white dashed circles indicate the nuclear LEC1 expression; e, exine; asterisk, site of exine rupture. The videos used for this figure can be found in Scale bar = 10 μm. Supporting Information.

**Movie S1A.** Transmission for Figure 1 (*LEC1:LEC1‐GFP* suspensorless embryo).
**Movie S1B.** Fluorescence for Figure 1 (*LEC1:LEC1‐GFP* suspensorless embryo).
**Movie S2A.** Transmission for Figure 2 (*DR5v2:nTdtomato* suspensorless embryo).
**Movie S2B.** Fluorescence for Figure 2 (*DR5v2:nTdtomato* suspensorless embryo).
**Movie S3A.** Transmission for Figure 3 (*LEC1:LEC1‐GFP* suspensor embryo).
**Movie S3B.** Fluorescence for Figure 3 (*LEC1:LEC1‐GFP* suspensor embryo).
**Movie S4A.** Transmission for Figure 4 (*DR5v2:nTdtomato* suspensor embryo).
**Movie S4B.** Fluorescence for Figure 4 (*DR5v2:nTdtomato* suspensor embryo).
**Movie S5A1.** Transmission for Figure 5A (*LEC1:LEC1‐GFP* embryogenic callus).
**Movie S5A2.** Fluorescence for Figure 5(A) (*LEC1:LEC1‐GFP* embryogenic callus).
**Movie S5B1.** Transmission for Figure 5(B) (*LEC1:LEC1‐GFP* embryogenic callus).
**Movie S5B2.** Fluorescence for Figure 5(B) (*LEC1:LEC1‐GFP* embryogenic callus).
**Movie S6A1.** Transmission for Figure 6(A) (*DR5v2:nTdtomato* embryogenic callus).
**Movie S6A2.** Fluorescence for Figure 6(A) (*DR5v2:nTdtomato* embryogenic callus).
**Movie S6B1.** Transmission for Figure 6(B) (*DR5v2:nTdtomato* embryogenic callus).
**Movie S6B2.** Fluorescence for Figure 6(B) (*DR5v2:nTdtomato* embryogenic callus).
**Movie S7A1.** Transmission for Figure S3(A) (*LEC1:LEC1‐GFP* arrested exine‐enclosed embryo).
**Movie S7A2.** Fluorescence for Figure S3(A) (*LEC1:LEC1‐GFP* arrested exine‐enclosed embryo).
**Movie S7B1.** Transmission for Figure S3(B) (*DR5v2:nTdtomato* arrested exine‐enclosed embryo).
**Movie S7B2.** Fluorescence for Figure S3(B) (*DR5v2:nTdtomato* arrested exine‐enclosed embryo).
**Movie S8A.** Transmission for Figure S4 (*LEC1:LEC1‐GFP* suspensorless embryo).
**Movie S8B.** Fluorescence for Figure S4 (*LEC1:LEC1‐GFP* suspensorless embryo).
**Movie S9A** Transmission for Figure S5 (*DR5v2:nTdtomato* suspensorless embryo).
**Movie S9B.** Fluorescence for Figure S5 (*DR5v2:nTdtomato* suspensorless embryo).
**Movie S10A.** Transmission for Figure S6 (*LEC1:LEC1‐GFP* suspensor embryo).
**Movie S10B.** Fluorescence for Figure S6 (*LEC1:LEC1‐GFP* suspensor embryo).
**Movie S11A1.** Transmission for Figure S7(A) (*LEC1:LEC1‐GFP* embryogenic callus).
**Movie S11A2.** Fluorescence for Figure S7(A) (*LEC1:LEC1‐GFP* embryogenic callus).
**Movie S11B1.** Transmission for Figure S7(B) (*LEC1:LEC1‐GFP* embryogenic callus).
**Movie S11B2.** Fluorescence for Figure S7(B) (*LEC1:LEC1‐GFP* embryogenic callus).

## Data Availability

Data available on request from the authors.
