# Supplementary material for: Establishment and maintenance of embryogenic cell fate during microspore embryogenesis
Source: Plant J. 2025 Feb 21;121(4):e17243. doi: 10.1111/tpj.17243 (PMC11843592; doi:10.1111/tpj.17243)

Supplementary Figure 1
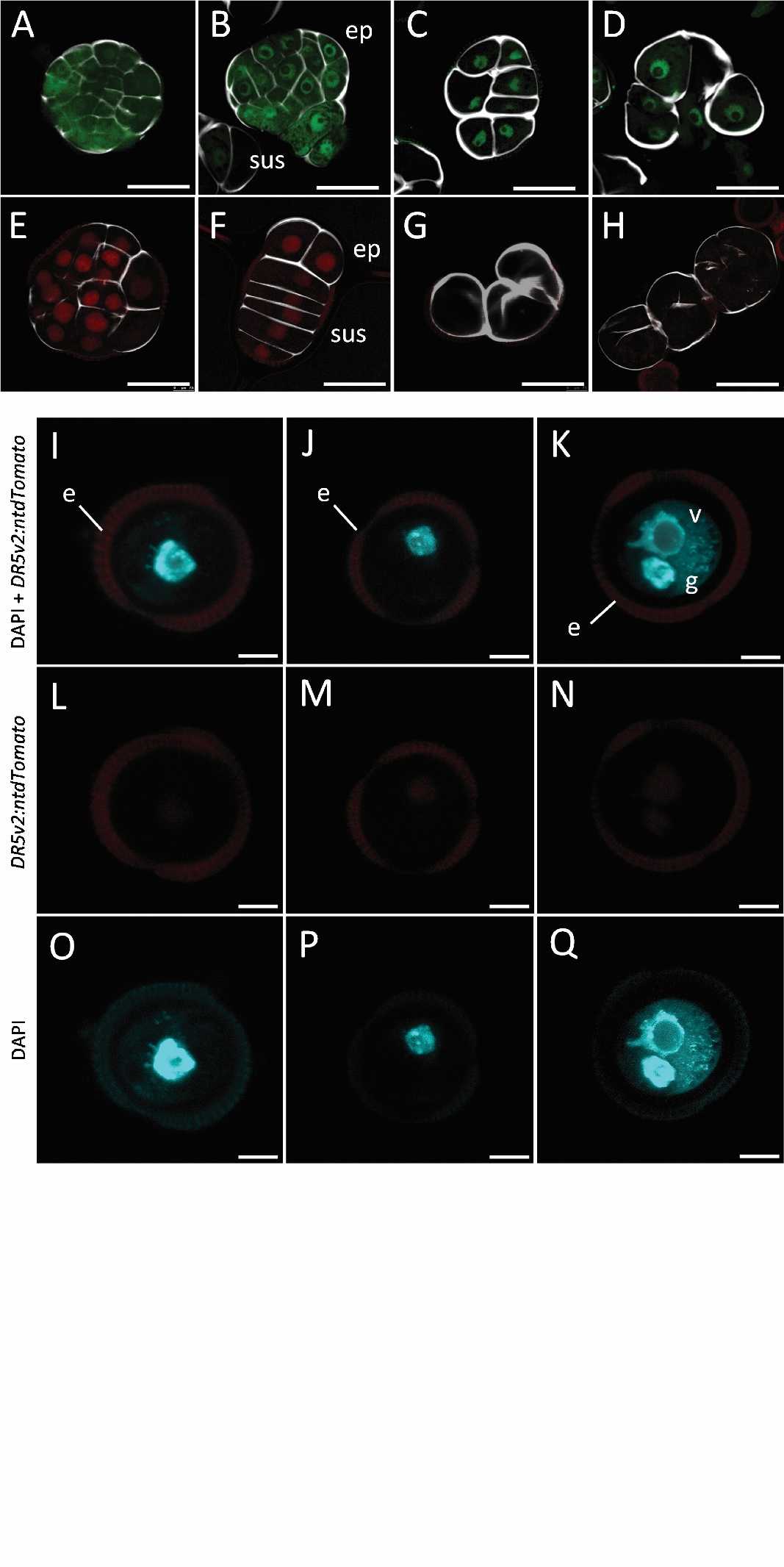


Supplementary Figure 2


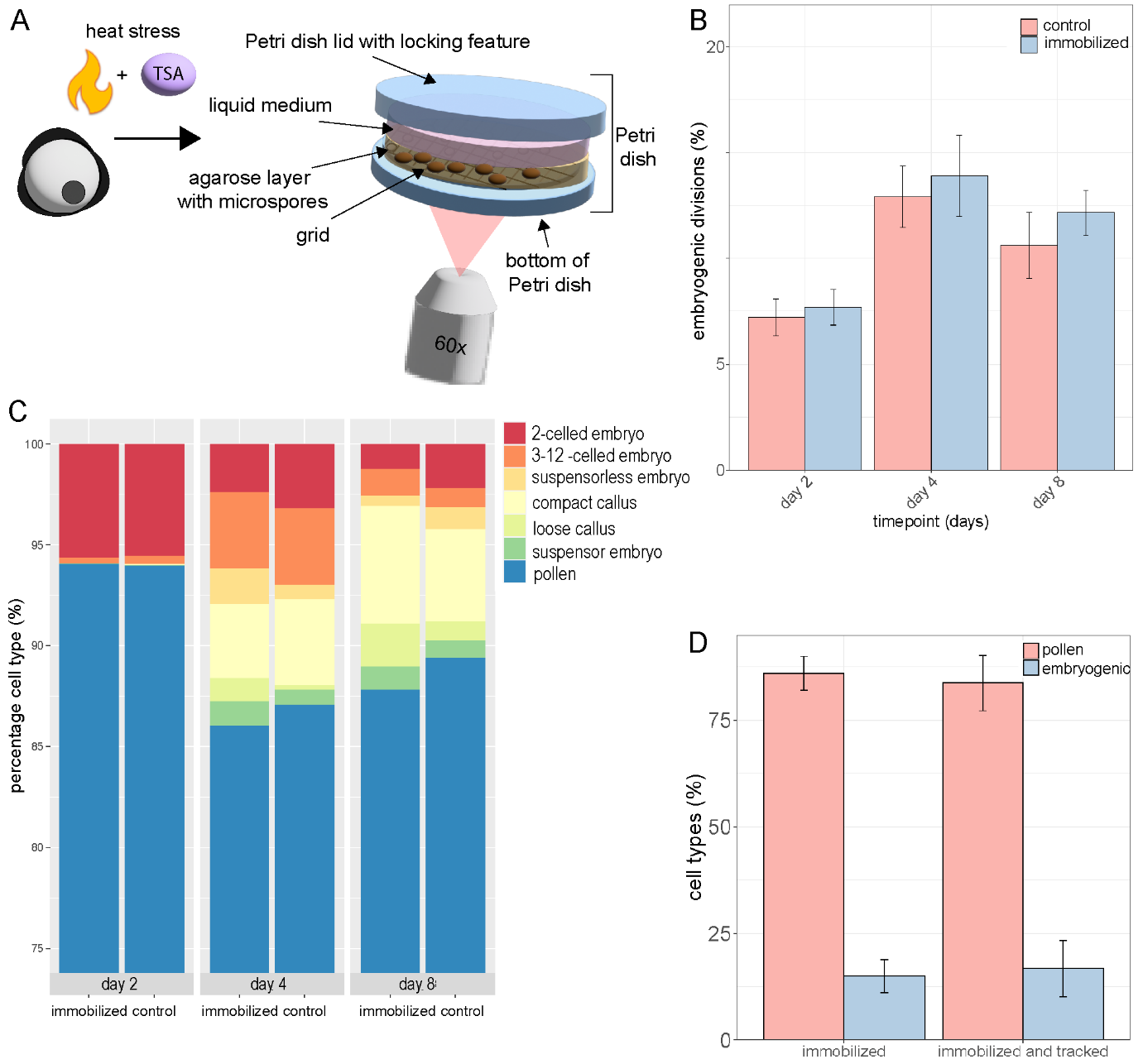


Supplementary Figure 3


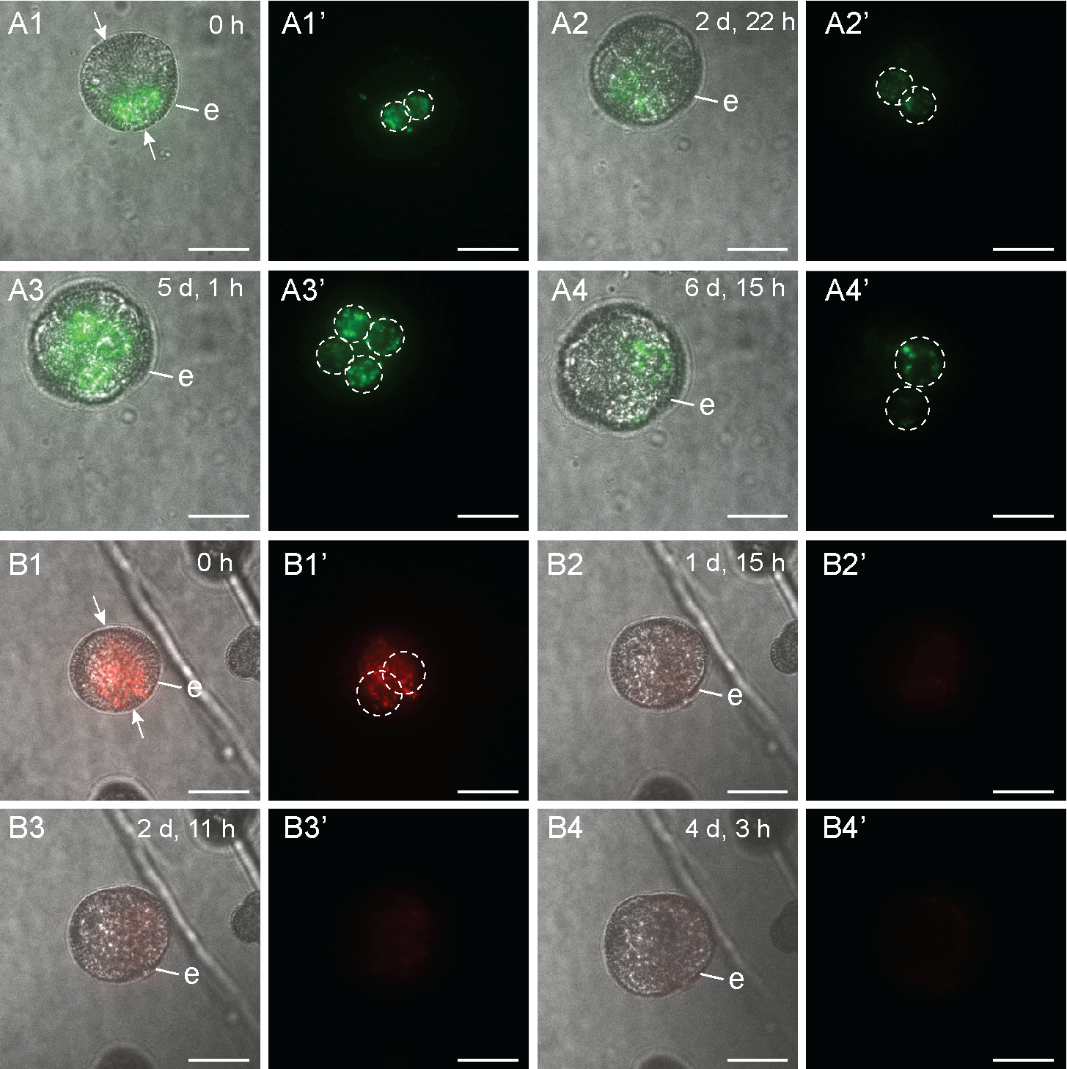


Supplementary Figure 4


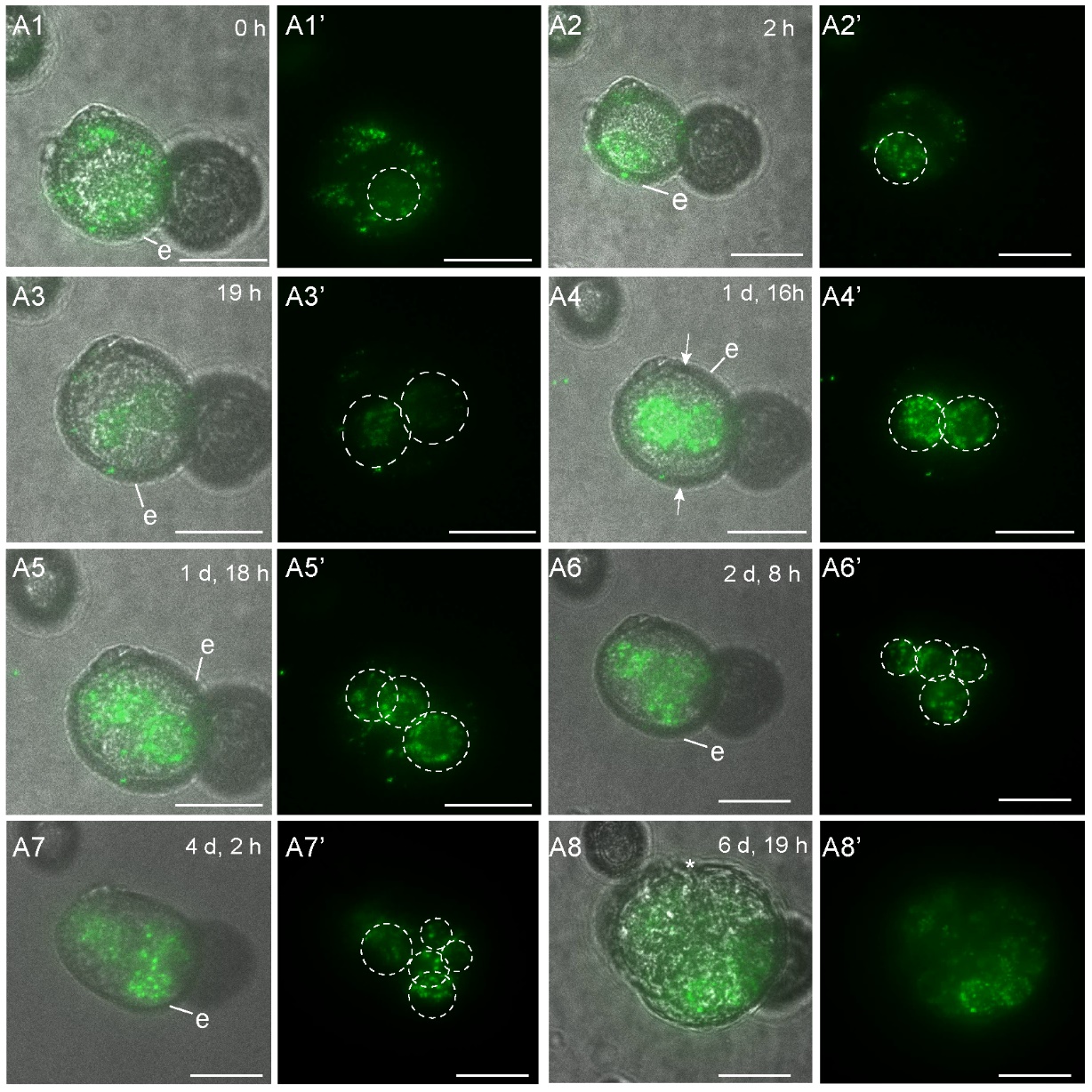


Supplementary Figure 5


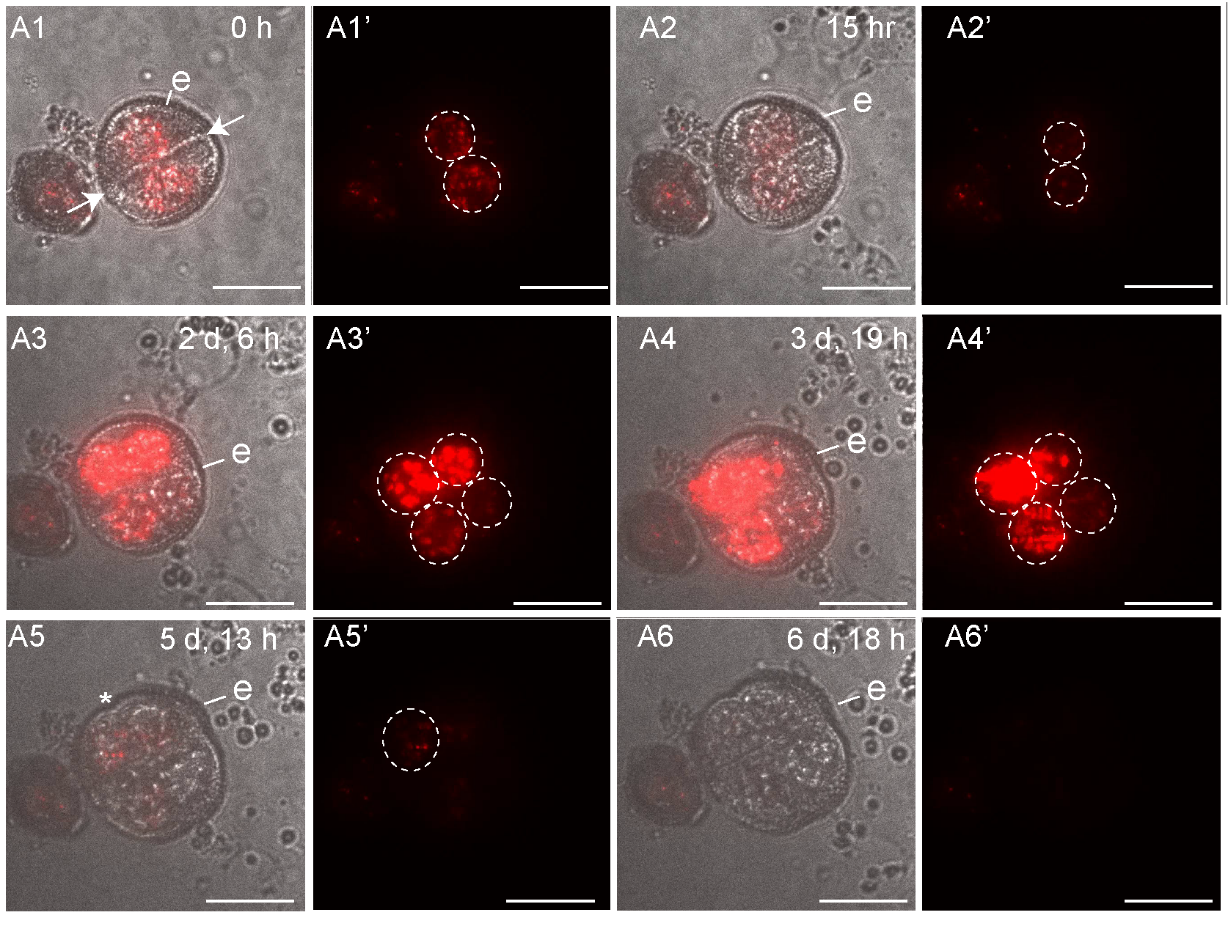


Supplementary Figure 6


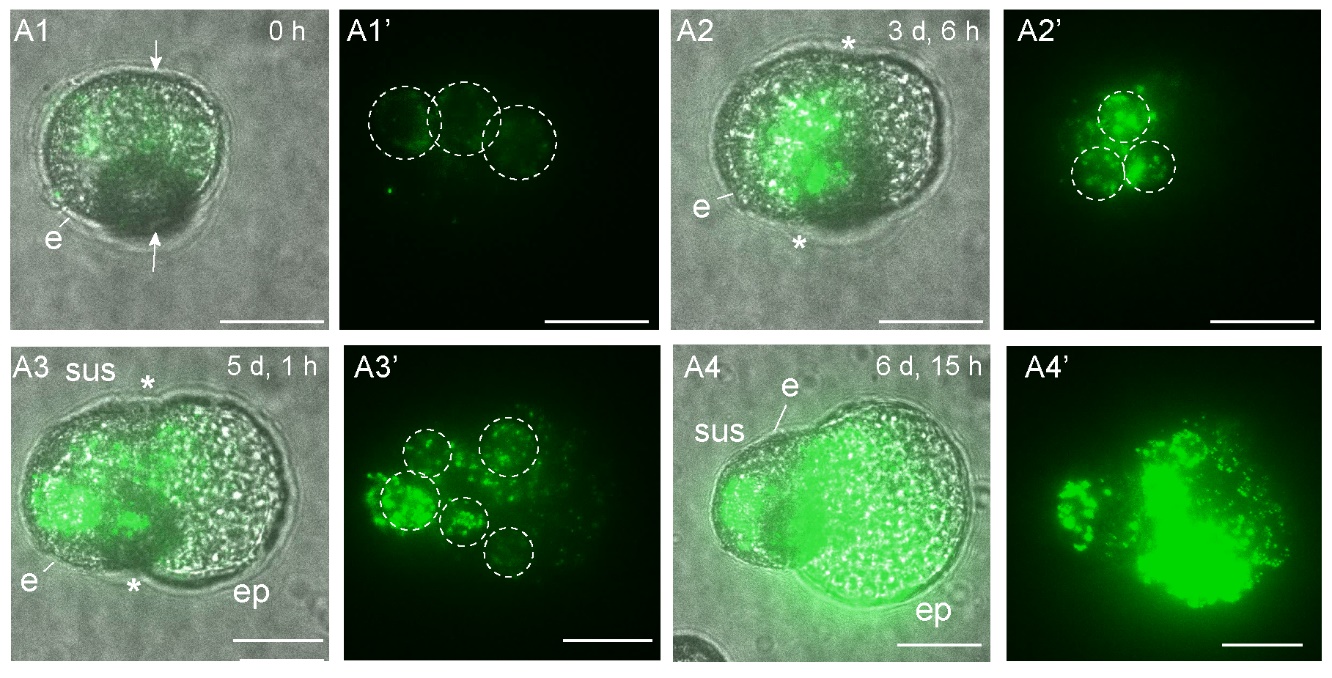


Supplementary Figure 7


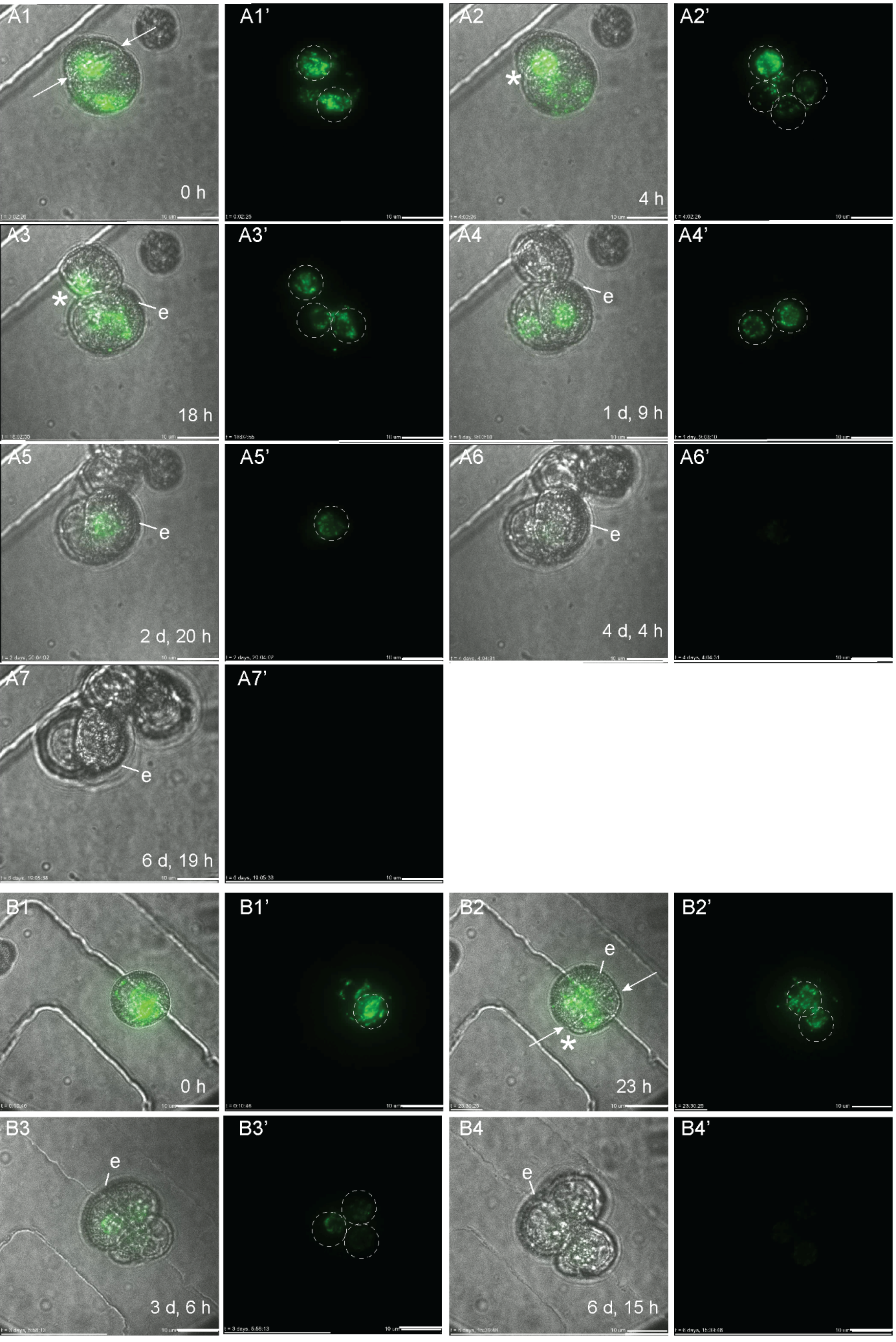

Supplement: Supplementary file 1 — Figure S1. Pollen and embryogenic structures found in microspore cultures. (A–H) Confocal laser scanning microscopy images of embryogenic structures at day 5 of culture expressing the LEC1:LEC1‐GFP (green fluorescence) (A–D) or DR5v2:ntdTomato (red fluorescence) reporters (E–H). White fluorescence, Renaissance SCR2200‐stained cell walls. (A, E) Suspensorless embryo after exine rupture; (B, F) suspensor‐bearing embryo after exine rupture; (C, G) compact callus; and (D, H) loose callus after exine rupture. (I–Q) Confocal laser scanning microscopy images of DAPI‐stained nuclei (blue) in gametophytic structures from a DR5v2:ntdTomato reporter line. All structures can be found at the start and during culture. No DR5v2:ntdTomato expression is observed. (I–O) Mid uninucleate microspore with the nucleus positioned in the middle of the cell. (J–P) Late uninucleate microspore just before pollen mitosis I, with the nucleus positioned close to the cell wall. (K–Q) Early bicellular pollen, with a larger vegetative nucleus (v) and a smaller generative nucleus (g). The exine (e) in I–Q shows autofluorescence in the light ranges used to detect DAPI and tdTomato. sus, suspensor; ep, embryo proper. Scale bars, A–H, 25 μm; I–Q, 5 μm. Figure S2. Time‐lapse imaging of microspore embryo cultures does not affect the viability or developmental fate of embryogenic structures. LEC1:LEC1‐GFP cultures were treated with HS + 0.05 μM TSA for 24 h, after which the cultures were immobilized in agarose (immobilized) or transferred to the same type of Petri dishes (Ibidi) with liquid medium only (control) before being transferred to 25°C for further culture. (A) Immobilization system. Microspores/pollen (indicated as brown ovals) were embedded in agarose in a Petri dish with a coverslip‐like bottom, forming at most two layers of cells, and then covered with a layer of liquid medium. (B) The effect of immobilization on embryogenic structure development. The percentage of embryogenic structures formed [file TPJ-121-0-s002.docx]
